# The Heaviest Bottleable Metallylone: Synthesis of a Monatomic, Zero‐Valent Lead Complex (“Plumbylone”)

**DOI:** 10.1002/anie.202209442

**Published:** 2022-08-08

**Authors:** Jian Xu, Sudip Pan, Shenglai Yao, Gernot Frenking, Matthias Driess

**Affiliations:** ^1^ Department of Chemistry: Metalorganics and Inorganic Materials Technische Universität Berlin Strasse des 17. Juni 115, Sekr. C2 10623 Berlin Germany; ^2^ Philipps-Universität Marburg Fachbereich Chemie 35032 Marburg Germany; ^3^ Donostia International Physics Center (DIPC) 20018 San Sebastian Spain

**Keywords:** Chelate Ligands, Lead, Plumbylone, Silylene, Tetrylone

## Abstract

The elusive plumbylone {[Si^II^(Xant)Si^II^]Pb^0^} **3** stabilized by the bis(silylene)xanthene chelating ligand **1**, [Si^II^(Xant)Si^II^=PhC(N*t*Bu)_2_Si(Xant)Si(N*t*Bu)_2_CPh], and its isolable carbonyl iron complex {[Si^II^(Xant)Si^II^]Pb^0^Fe(CO)_4_} **4** are reported. The compounds **3** and **4** were obtained stepwise via reduction of the lead(II) dibromide complex {[Si^II^(Xant)Si^II^]PbBr_2_} **2**, prepared from the bis(silylene)xanthene **1** and PbBr_2_, employing potassium naphthalenide and K_2_Fe(CO)_4_, respectively. While the genuine plumbylone **3** is rather labile even at −60 °C, its Pb^0^→Fe(CO)_4_ complex **4** turned out to be relatively stable and bottleable. However, solutions of **4** decompose readily to elemental Pb and {[Si^II^(Xant)Si^II^]Fe(CO)_3_} **5** at 80 °C. Reaction of **4** with [Rh(CO)_2_Cl]_2_ leads to the formation of the unusual dimeric [(OC)_2_RhPb(Cl)Fe(CO)_4_] complex **6** with trimetallic Rh−Pb−Fe bonds. The molecular and electronic structures of **3** and **4** were established by Density Functional Theory (DFT) calculations.

## Graphical abstract

The elusive plumbylone {[Si^II^(Xant)Si^II^]Pb^0^} stabilized by a bis(silylene)xanthene chelating ligand, [Si^II^(Xant)Si^II^=PhC(N*t*Bu)_2_Si(Xant)Si(N*t*Bu)_2_CPh], and its isolable carbonyl iron complex {[Si^II^(Xant)Si^II^]Pb^0^Fe(CO)_4_} are reported. The latter can insert the PbFe(CO)_4_ moiety into a Rh−Cl bond to give a RhPbClFe(CO)_4_ complex.

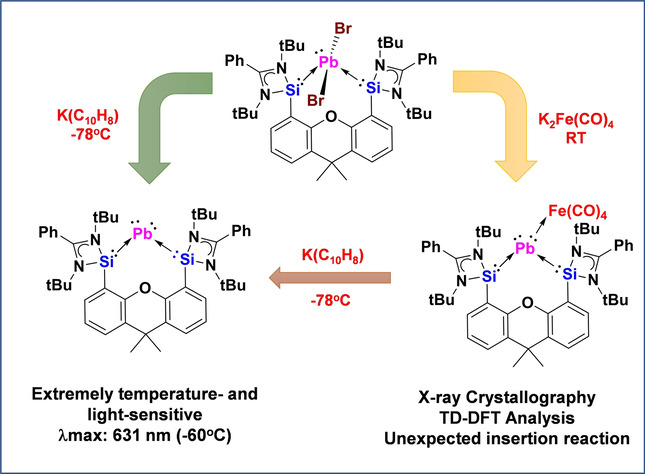

Zero‐valent Group 14 element complexes have attracted considerable attention by main‐group and organometallic chemists since Frenking and co‐workers in 2006 reinterpreted the unusual C−P bonding involved in carbodiphosphorane C(PPh_3_)_2_ as Lewis donor‐acceptor interactions.^[1]^ The name tetrylone (C: carbone; Si: silylone; Ge: germylone; Sn: stannylone; Pb: plumbylone) was given to this new type of compounds with the general form L:→E^0^←:L (L=σ‐donor, E=C, Si, Ge, Sn, Pb) following the theoretical calculations.^[2]^ Since then, several series of tetrylones have been isolated and have been considered as a soluble “allotrope” of the respective elements.^[3]^ Remarkable examples include the *N*‐heterocyclic carbene (NHC) stabilized carbone **A**,^[4]^ the cyclic (alkyl)(amino)carbene supported silylone and germylone **B**,[^5^, ^6^] and the bis‐NHCs stabilized silylone and germylone **C** (Scheme 1).[^7^, ^8^] Compared to carbones, isolable silylones, germylones and stannylone are still scarce. Despite DFT calculations had predicted that NHC adducts of Pb^0^ may be synthetically accessible,^[9]^ all previous attempts failed.^[10]^ To date, no monoatomic zero‐valent lead compound has been reported, presumably, due to their intrinsic kinetic lability and sensitivity to light, which facilitates decomposition into ‘free’ ligands and elemental lead.

**Scheme 1:**
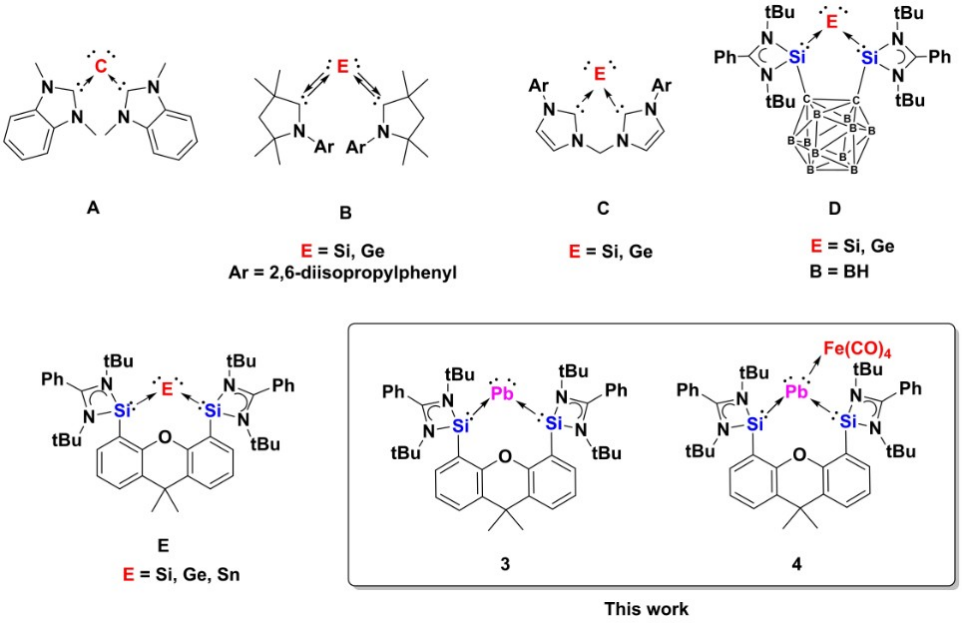
Examples of the tetrylones **A**–**E** and the zero‐valent lead complexes **3** and **4** of this work.

*N*‐heterocyclic silylenes (NHSis) with a three‐coordinated silicon(II) center (intramolecularly imino donor‐stabilized) are stronger σ‐donor ligands with respect to NHCs. Taking advantage of the chelating effect and the electron‐richness of bis‐NHSis, we have successfully developed two series of isolable silylones and germylones **D**[^11^, ^12^] and **E**[^13^, ^14^] (E=Si, Ge; Scheme 1), respectively. Using the xanthene‐based bis‐NHSis, we could even realize the bottleable two‐coordinate stannylone **E** ^[15]^ (E=Sn; Scheme 1) quite recently. Encouraged by the successful synthesis of **E**, we set out our vision for extending our tetrylone chemistry to the heaviest Group 14 element, lead, by utilizing the same bis‐NHSis ligand. Herein, we report the synthesis, characterization, and electronic structure of the first plumbylone {[Si^II^(Xant)Si^II^]Pb^0^} **3** [Si^II^(Xant)Si^II^=PhC(N*t*Bu)_2_Si(Xant)Si(N*t*Bu)_2_CPh] and its tetracarbonyl iron complex {[Si^II^(Xant)Si^II^]Pb^0^Fe(CO)_4_} **4** supported by bis(silylene)xanthene (Scheme 1). The structures of **2**, **4**, **5** and **6** were confirmed by X‐ray crystallographic studies.^[16]^

Treatment of bis(silylene)xanthene [Si^II^(Xant)Si^II^] **1** ^[17]^ with one molar equiv. of PbBr_2_ in THF at room temperature yields {[Si^II^(Xant)Si^II^]PbBr_2_} **2** as a pale yellow powder in 87 % yields after workup (Scheme 2). The latter was characterized by standard spectroscopic methods, and its molecular structure is established by a single‐crystal X‐ray diffraction analysis (XRD) (Figure 1). The ^29^Si NMR spectrum of **2** exhibits a singlet at *δ*=105.0 ppm, which is significantly down‐field shifted compared to that of the tin homologue (30.3 ppm). However, the molecular structure of **2** is isostructural to that of the tin homologue,^[15]^ featuring a lead center adopting a see‐saw geometry with the two bromine atoms located in the axial positions (Br1−Pb1−Br2: 167.55(1)°). No ^207^Pb NMR resonance could be observed due to quadrupolar ^79/81^Br nuclei which drastically broaden the signal.^[18]^

**Scheme 2:**
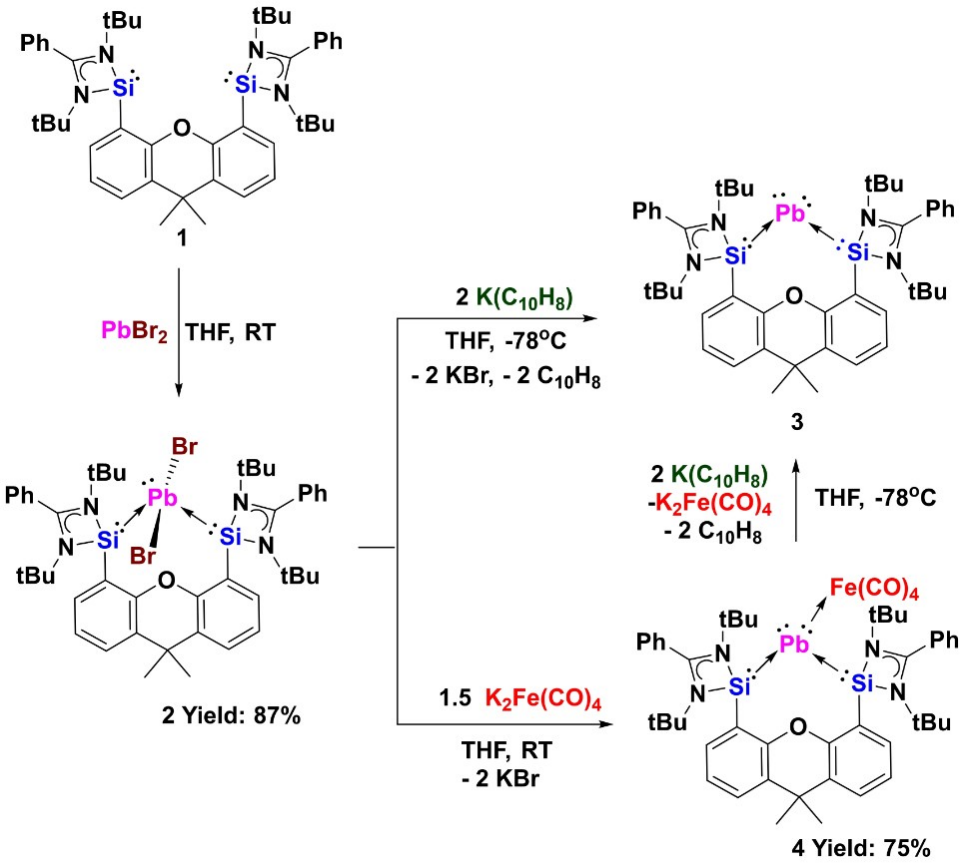
Synthesis of the bis(silylene)xanthene supported plumbylone **3** and **4** via the PbBr_2_ complex **2**.

**Figure 1:**
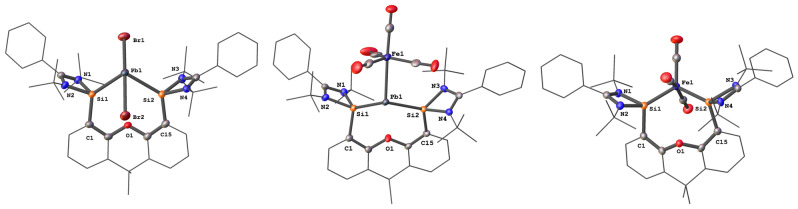
Molecular structure of **2**, **4** and **5**.^[16]^ Thermal ellipsoids are drawn at the 50 % probability level. H atoms and solvent molecules are omitted for clarity.

With the Pb^II^ halide precursor **2** in hand, we envisioned that the desired plumbylone could be obtained through its reductive debromination. To our delight, the reaction of **2** with two molar equivs. of freshly prepared potassium naphthalenide (K(C_10_H_8_)) led to an intensive blue solution of **3** in THF at −78 °C, indicating the formation of the expected plumbylone {[Si^II^(Xant)Si^II^]Pb^0^} **3**. At −60 °C, the UV/Vis spectrum of the latter blue solution recorded in THF displays an absorption at *λ*=631 nm, which is quite close to the absorption (674 nm) observed for the isolable stannylone analogue **E** (E=Sn). Unfortunately, **3** is rather fragile in solutions and undergoes decomposition into elemental lead and “free” ligand **1** even at −50 °C, up to now, all attempts to isolate **3** failed.

Considering our previous success of synthesizing the bis(silylene)pyridine supported germylone carbonyl iron complex {[Si(Py)Si]Ge^0^Fe(CO)_4_}^[19]^ and bis(silylene)xanthene stabilized stannylone carbonyl diiron complex {[Si^II^(Xant)Si^II^]Sn^0^[Fe(CO)_4_]_2_},^[15]^ introduction of Lewis acidic Fe(CO)_4_ could increase the stability of the elusive plumbylone **3**. We thus conducted the reaction of **2** with 1.5 molar equivs. of Collman's reagent [K_2_Fe(CO)_4_]^[20]^ in THF. In fact, the latter reaction allowed us to form and isolate the first plumbylone complex {[Si^II^(Xant)Si^II^]Pb^0^Fe(CO)_4_} **4** as a red powder in 75 % yields. Its ^1^H NMR spectrum shows two singlets at *δ*=1.09 and 1.23 ppm for the tert‐butyl groups, implying an asymmetric structure. The ^29^Si NMR spectrum of **4** displays a singlet at *δ*=16.6 ppm, which is reminiscent of the ^29^Si NMR chemical shift of {[Si^II^(Xant)Si^II^]Sn^0^[Fe(CO)_4_]_2_} (*δ*=27.0 ppm).^[15]^ The ^207^Pb NMR spectrum of the plumbylone complex **4** exhibits a singlet at *δ*=2238.9 ppm. The IR spectrum of **4** shows CO stretching vibrations (νCO) at 1951, 1869, 1848 and 1831 cm^−1^, which are significantly red‐shifted compared to the values of PbFe(CO)_4_ (2049, 2006, 1990, 1978 and 1964 cm^−1^).^[21]^ The molecular structure of **4** reveals a Pb^0^ atom with a trigonal‐pyramidal coordination geometry, implying that a lone pair of electrons occupy the apex while the other lone pair coordinates to the Fe(CO)_4_ moiety (Figure 1). The Pb−Si distances of 2.7948(8) and 2.7914(7) Å are slightly longer than those in its precursor **2** (2.7594(9) and 2.775(1) Å). The Pb−Fe distance of 2.7367(5) Å is similar to those values for the dimer of [Et_2_PbFe(CO)_4_].^[22]^

As the stannylone {[Si^II^(Xant)Si^II^]Sn^0^} was obtained by us via reduction of {[Si^II^(Xant)Si^II^]Sn^0^[Fe(CO)_4_]_2_},^[15]^ we also performed the reduction of **4** with different reducing reagents. Using potassium naphthalenide, we observed again the characteristic blue color of {[Si^II^(Xant)Si^II^]Pb^0^} **3** (Scheme 2). However, isolation of this genuine plumbylone remained challenging owing to its intrinsic lability.

In order to shed light on the structural and electronic properties of the plumbylones, DFT calculations of compounds **3** and **4** were performed at the BP86‐D3(BJ)/def2‐TZVP level (see Supporting Information for details). The calculated structural parameters of **4** closely match the geometry of the XRD analysis (Table S4 and Figure S23). The optimized structure of **3** is shown in Figure 2a. The calculated Si−Pb bonds in **3**, which differ slightly due to steric interactions of the bulky substituents, are significantly shorter (2.630–2.633 Å) than in **4** (2.750–2.759 Å). The bond lengths concur with the standard value for a covalent Si−Pb single bond (2.60 Å).^[23]^ Dative bonds are usually longer than electron‐sharing bonds,^[24]^ but the Si:→Pb donor bond is enhanced by some Pb:→Si π‐backdonation (s. below) which yields partial double‐bond character. The Wiberg bond orders of the Si−Pb bonds is 1.12/1.14. The lead atom is strongly bonded to the chelating ligand **1** with a calculated bond dissociation energy of *D*_e_=77.0 kcal mol^−1^ (Δ*G* ^298^=66.1 kcal mol^−1^).

**Figure 2:**
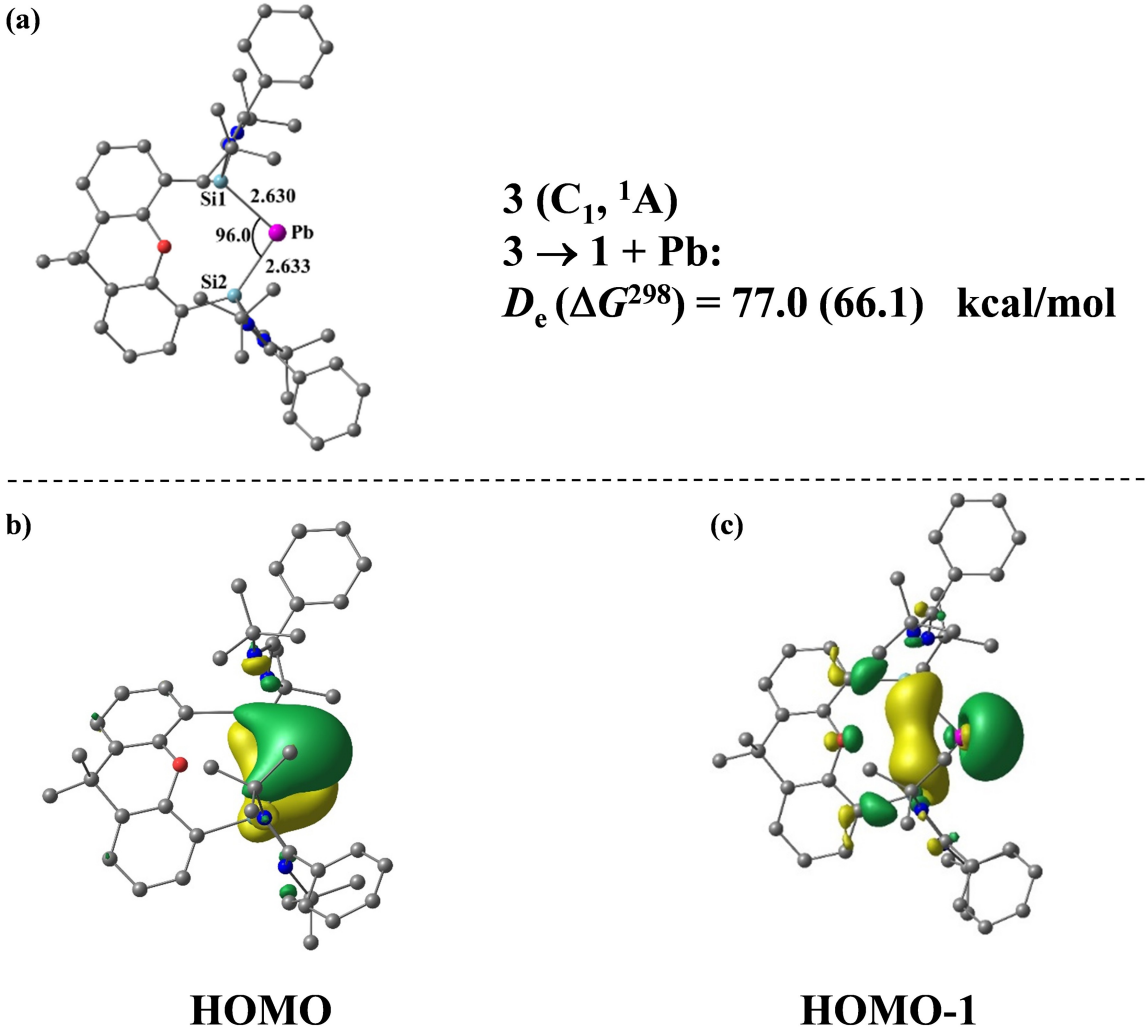
a) Calculated equilibrium geometry of **3** with the most important bond lengths [Å] and angles [°]. b) HOMO of **3**. c) HOMO−1 of **3**.

Inspection of the HOMO and HOMO‐1 displayed in Figures 2b, c shows the characteristic features of an ylidone L:→E^0^←:L with π‐ and σ‐lone pair orbitals at Pb and some backdonation to the ligands. The assignment as a Pb^0^ compound for **3** is supported by the atomic partial charges of the NBO method, which suggests a negative value of −0.37 e for Pb although lead is less electronegative (1.8) than silicon (1.9). The bonding situation is further supported by EDA‐NOCV calculations, which indicate that the orbital interactions between Pb^0^ and the ligand are dominated by in‐phase (+,+) and out‐of‐phase (+,−) donation L:→Pb^0^←:L, providing 40 % (Δ*E*_orb(1)_) and 30 % (Δ*E*_orb(2)_) of the orbital term, respectively, while the backdonation L:←Pb^0^→:L yields 22 % (Δ*E*_orb(3)_)_._ The numerical results of the EDA‐NOCV calculations and the associated deformation densities of the orbital terms are given in Table S9 and Figure S24. We calculated the UV/Vis spectra of **3** and **4** to identify the bare plumbylone. The theoretical spectra of **3** and **4** shown in Figure S25 and S26 are in very good agreement with the experimental findings. The large shift of the highest lying peak of **4** (431 nm) to **3** (631 nm) by 200 nm closely matches the calculated shift from **4** (389 nm) to **3** (609 nm) by 220 nm, which leaves no doubt that the experimentally observed species is the Pb^0^ species.

The ylidones (L:→E^0^←:L) exhibit rather large first and second proton affinities due to the existence of two lone pair orbitals.^[25]^ Table S10 shows that the calculated first (277.0 kcal mol^−1^) and second (176.7 kcal mol^−1^) proton affinities of **3** at RT are very high. The calculations predict that the plumbylone binds also one (21.7 kcal mol^−1^) and two BH_3_ ligands (18.0 kcal mol^−1^) quite strongly. The Fe(CO)_4_ fragment in complex **4** has a calculated bond strength at RT of 63.4 kcal mol^−1^. A second Fe(CO)_4_ ligand is calculated with a bond strength of 36.2 kcal mol^−1^ (Table S10) but could not be observed under the present reaction conditions.

In the solid state, complex **4** (m.p. 156 °C (decomp.)) is stable and can be stored in a glove box for months. However, in solutions, **4** is thermolabile and light sensitive. It decomposes rapidly to elemental lead at ca. 80 °C, generating carbonyl iron moieties which can be re‐coordinated by the bis(silylene)xanthene **1** to afford the new bis‐NHSis‐supported Fe(CO)_3_ complex **5** (Scheme 3). Compound **5** has been characterized by ^1^H‐, ^13^C{^1^H}‐, and ^29^Si{^1^H} NMR spectroscopy (see Supporting Information), and its molecule structure was confirmed by XRD (Figure 1). A related bis(silylene)pyridine Fe(CO)_3_ complex has been descripted by our group previously.^[26]^

**Scheme 3:**
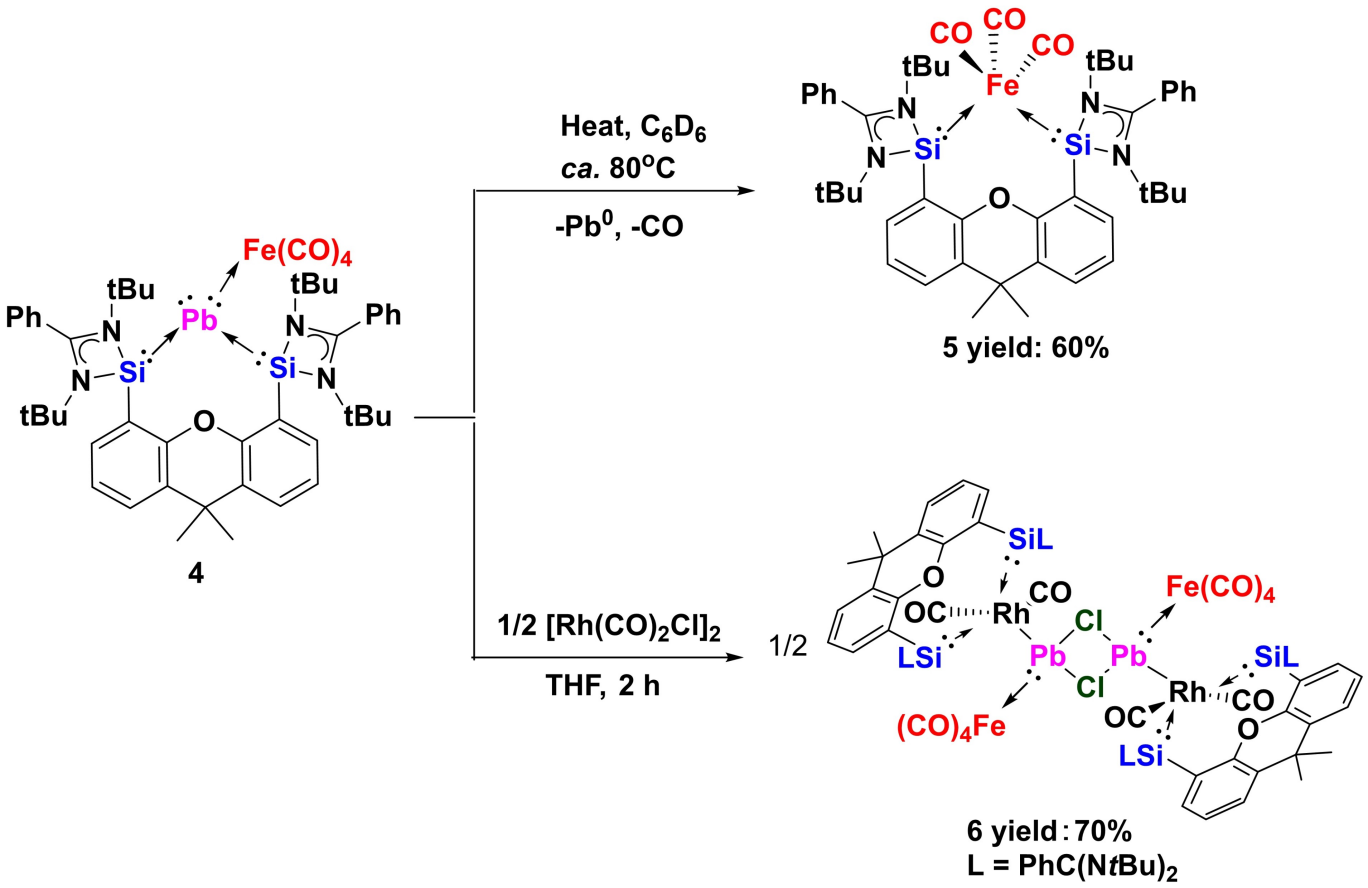
Synthesis of **5** and **6**.

Considering the electron‐rich zero‐valent Pb^0^ center of **4** with two lone electron pairs, one already coordinate with Fe(CO)_4_, the other remaining potentially active towards Lewis‐acid sites, we mixed **4** with 0.5 molar equivs. of [Rh(CO)_2_Cl]_2_ in THF. Surprisingly, **4** underwent an unexpected insertion reaction of the PbFe(CO)_4_ moiety into the Rh−Cl bond to give the dimeric (OC)_2_RhPb(Cl)Fe(CO)_4_ complex **6** as a dark brown solid in 70 % isolated yields (Scheme 3). The ^29^Si NMR spectrum of **6** displays a doublet at *δ*=77.45 ppm (*J*_Rh‐Si_=63.99 Hz). Similar to **2**, no ^207^Pb NMR singnal of **6** could be detected, which accords with the same reported problems of organolead(II) halides.^[27]^ Its molecular structure reveals a dimer symmetrically bridged by two chlorine atoms (Figure 3). The bis(silylene)xanthene‐chelated Rh atom is directly bonded to the Pb atom of PbFe(CO)_4_ and adopts a trigonal‐bipyramidal geometry. As a result, the four‐coordinate lead atom possesses a distorted tetrahedral coordination environment with a Pb−Rh distance of 2.6943(2) Å, which is slightly shorter than the Rh−Pb dative bond observed in CpRh(PMe_2_Ph)_2_⋅PbCl_2_ (2.7561(7) Å).^[28]^ It should be noted that **6** represents a very rare example containing a trimetallic Rh−Pb−Fe bond.[^29^, ^30^, ^31^]

**Figure 3:**
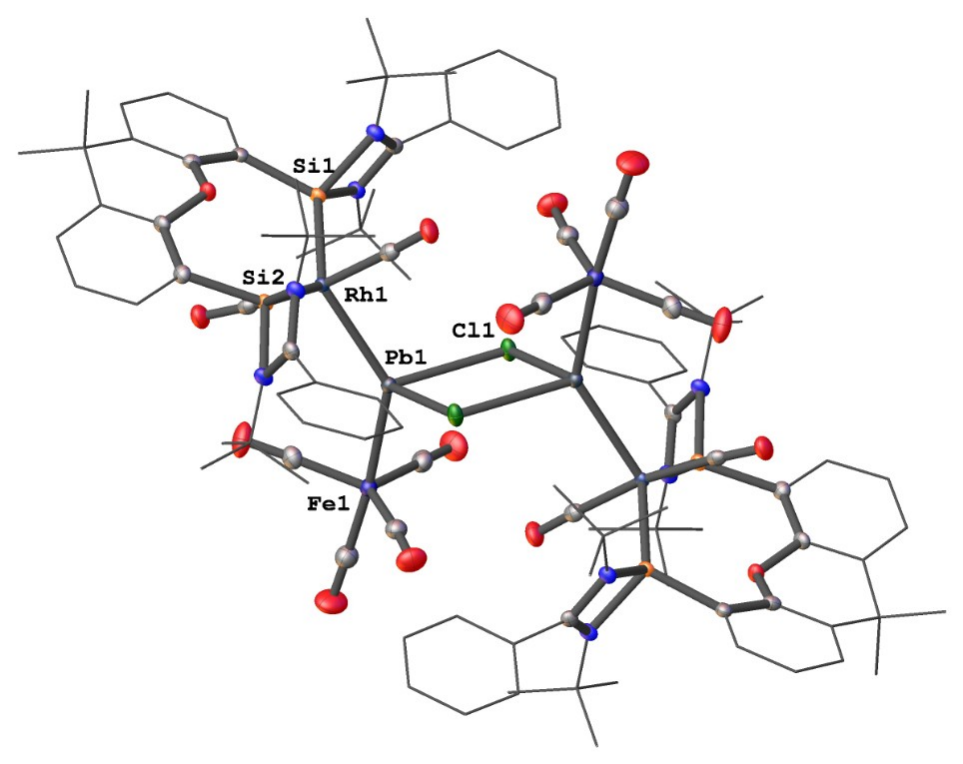
Molecular structure of **6**.^[16]^ Thermal ellipsoids are drawn at the 50 % probability level. H atoms and solvent molecules are omitted for clarity.

In summary, we were able to synthesize the elusive plumbylone {[Si^II^(Xant)Si^II^]Pb^0^} **3** and could stabilize it through carbonyl iron complexation to give {[Si^II^(Xant)Si^II^]Pb^0^Fe(CO)_4_} **4**. Unlike the “lighter” Si, Ge and Sn analogues, the plumbylone **3** is extremely temperature‐ and light‐sensitive. The PbFe(CO)_4_ complex **4** decomposes at much higher temperature (ca. 80 °C) to form elemental Pb and the corresponding bis(silylene) Fe(CO)_3_ complex. The structural and electronic properties of **3** and **4** have been investigated with DFT and TD‐DFT calculations. As expected, the HOMO and HOMO‐1 of **3** correspond to a π‐type and a σ‐type lone pair, respectively. Furthermore, insertion reaction of **4** with [Rh(CO)_2_Cl]_2_ furnished an unusual dimeric [(OC)_2_RhPb(Cl)Fe(CO)_4_] complex **6**.

## Conflict of interest

The authors declare no conflict of interest.

## Supporting information

As a service to our authors and readers, this journal provides supporting information supplied by the authors. Such materials are peer reviewed and may be re‐organized for online delivery, but are not copy‐edited or typeset. Technical support issues arising from supporting information (other than missing files) should be addressed to the authors.

Supporting Information

## Data Availability

The data that support the findings of this study are available in the Supporting Information of this article.
