# Supplementary material for: The Heaviest Bottleable Metallylone: Synthesis of a Monatomic, Zero‐Valent Lead Complex (“Plumbylone”)
Source: Angew Chem Int Ed Engl. 2022 Aug 8;61(38):e202209442. doi: 10.1002/anie.202209442 (PMC9545849; doi:10.1002/anie.202209442)
Supplement: Supplementary file 1 — Supporting Information [file ANIE-61-0-s001.pdf]

Angewandte Chemie
Eine Zeitschrift der Gesellschaft Deutscher Chemiker

Supporting Information

The Heaviest Bottleable Metallylone: Synthesis of a Monatomic, Zero-Valent Lead Complex ("Plumbylone")

J. Xu, S. Pan, S. Yao, G. Frenking, M. Driess**

Supporting Information

Contents

A. Experimental Procedures.. 2
A1. General Considerations.. 2
A2. Single-Crystal X-ray Structure Determination.. 2
A3. Synthesis and Characterization.. 2
A4. X-ray Crystallographic Data.. 15
B. Theoretical Calculations.. 23
References.. 30

A. Experimental Procedures

A1. General Considerations

All experiments were carried out under dry oxygen-free nitrogen using standard Schlenk techniques or MBraun glove box fitted with a gas purification and recirculation unit. Solvents were dried by standard methods and freshly distilled prior to use. Bis(NHSi)xanthene Si^{II}(Xant)Si^{II} **1** [Si^{II}(Xant)Si^{II} = PhC(N*t*Bu)$_2$Si(Xant)Si(N*t*Bu)$_2$CPh],[1] and $K_2Fe(CO)_4$[2] were synthesized according to reported procedures. The solution NMR spectra were recorded on Bruker Spectrometers AV 200, 400 or 500 with residual solvent signals as internal reference (^{1}H NMR: Benzene-*d*$_6$, 7.16 ppm, THF-*d*$_8$, 3.58 and 1.72 ppm; ^{13}C{^{1}H} NMR: Benzene-*d*$_6$, 128.06 ppm, THF-*d*$_8$: 67.21 and 25.31 ppm) or external standards (^{29}Si{^{1}H} NMR: $SiMe_4$, 0.0 ppm; ^{207}Pb NMR: $Pb(NO_3)_2$ in D_2O, -2965.72 ppm). The following abbreviations were used to describe peak patterns when appropriate: *br* = broad, *s* = singlet, *d* = doublet, *t* = triplet, *dd* = doublet of doublets, *m* = multiplet. Elemental analyses were performed by the analytical labor service in the Institute of Chemistry, Technical University of Berlin, Germany. High-resolution ESI-MS were measured on a Thermo Scientific LTQ orbitrap XL. UV/Vis spectra were recorded on an Analytik Jena Specord S600 diode array spectrometer. IR spectra were measured with a Nicolet iS5 FT-IR-Spectrometer from the company Thermo.

A2. Single-Crystal X-ray Structure Determination

Crystals were each mounted on a glass capillary in perfluorinated oil and measured in a cold N_2 flow. The data of all compounds were collected on an Oxford Diffraction SuperNova, Single source at offset, Atlas at 150 K (Cu-Kα radiation, λ = 1.54184 Å). The structures were solved by direct methods and refined on F^2 with the SHELX-97 software package.[3] For the crystal of compounds **2** and **5**, the strongly disordered THF (**2**) and C_6H_6 (**5**) molecules were treated using Solvent Masking in Olex2. In the molecular structure of compound **4**, the lead atom is disordered over two positions with an approximate occupancy ratio of 0.98:0.02. **CCDC:** 2172109 (**2**), 2172106 (**4**), 2172108 (**5**) and 2172107(**6**) contain the supplementary crystallographic data for this paper. These data can be obtained free of charge from The Cambridge Crystallographic Data Centre via www.ccdc.cam.ac.uk/structures/

A3. Synthesis and Characterization

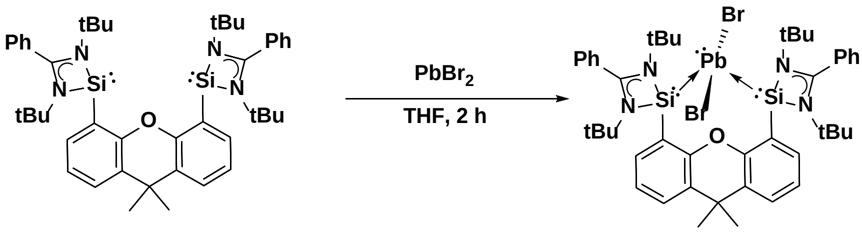

1

2 87% yield

Synthesis of Compound 2. To the mixture of bis(N-heterocyclic silylene) xanthene **1** (728 mg, 1 mmol) and $PbBr_2$ (367 mg, 1 mmol) in a 50 mL Schlenk flask was added 30 mL THF at room temperature under stirring. After stirring 2 h, volatiles were removed under vacuum and the residue was washed with Et_2O (20 mL) to afford compound **2** as a yellow powder after dried under vacuum (935 mg, 87% isolated yield). Yellow needle crystals suitable for X-ray single crystal diffraction analysis were obtained from a concentrated toluene solution at -30°C.

M.p. 195°C (decomp.). ^{1}H NMR (400 MHz, THF-*d*$_8$) *δ* = 7.95 – 7.90 (m, 2 H, Ar(Xant)-*H*), 7.73 – 7.65 (m, 4 H, Ar(Ph)-*H*), 7.63 – 7.58 (m, 6 H, Ar(Ph)-*H*), 7.54 (m, 2 H, Ar(Xant)-*H*), 7.23 (t, *J* = 7.4 Hz, 2 H, Ar(Xant)-*H*), 1.62 (s, 6 H, C(C*H*$_3$)$_2$), 1.27 (s, 36 H, NC(C*H*$_3$)$_3$). ^{13}C{^{1}H} NMR (101 MHz, THF-*d*$_8$) *δ* = 173.22 (s, NCN), 156.82, 132.73, 132.05, 131.49, 131.38, 131.20, 130.04, 129.18, 129.15, 128.50, 124.97, 124.17 (s, Ar-*C*), 55.54 (s, N*C*(CH$_3$)$_3$), 35.68 (s, *C*(CH$_3$)$_2$), 31.98 (s, NC(*C*H$_3$)$_3$), 31.43 (s, C(*C*H$_3$)$_2$). ^{29}Si{^{1}H} NMR (79 MHz, THF-*d*$_8$) *δ* = 105.00 (s). HRMS(ESI): (m/z) calcd for $[M\text{-}Br]^+$ ($(C_{45}H_{58}N_4OSi_2PbBr)^+$: 1013.3093; found: 1013.3083. Elemental analysis calcd for $C_{45}H_{58}N_4OSi_2PbBr_2 \cdot Et_2O$：C, 50.38; H, 5.87; N, 4.80 found: C, 45.62; H, 5.39; N, 4.46 [Consistently low C analysis may be due to the formation of silicon carbide].

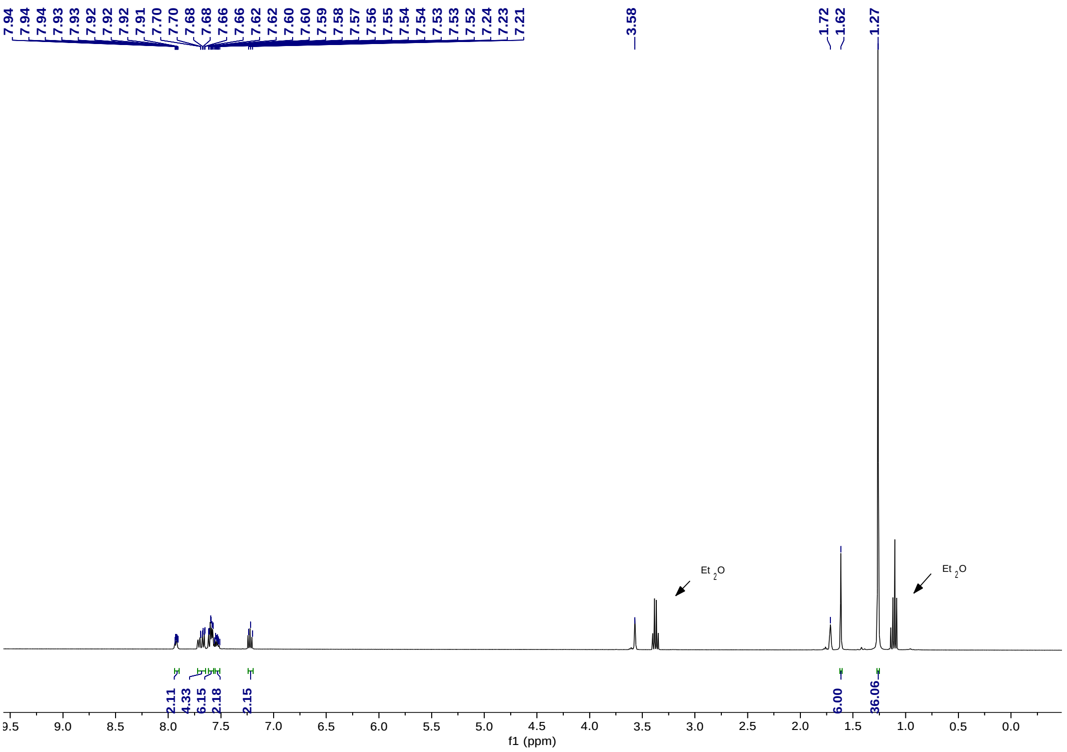

Figure S1. ^{1}H NMR spectrum of **2** in THF-d_8.

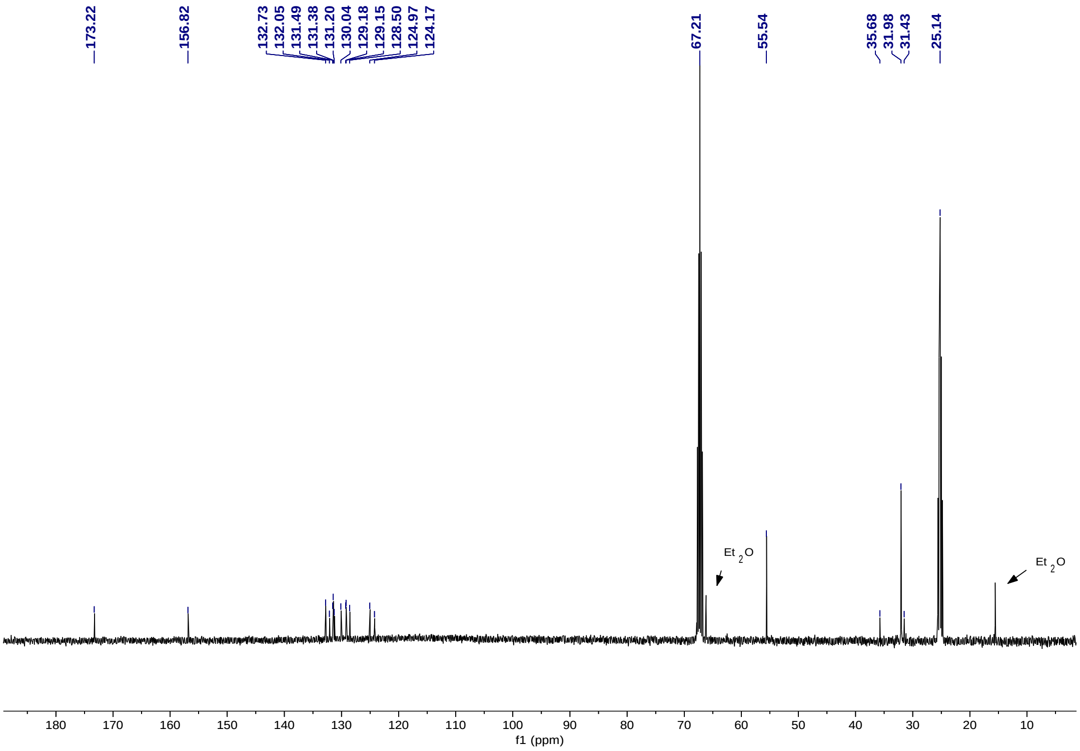

Figure S2. ^{13}C{^{1}H} NMR spectrum of **2** in THF-d_8.

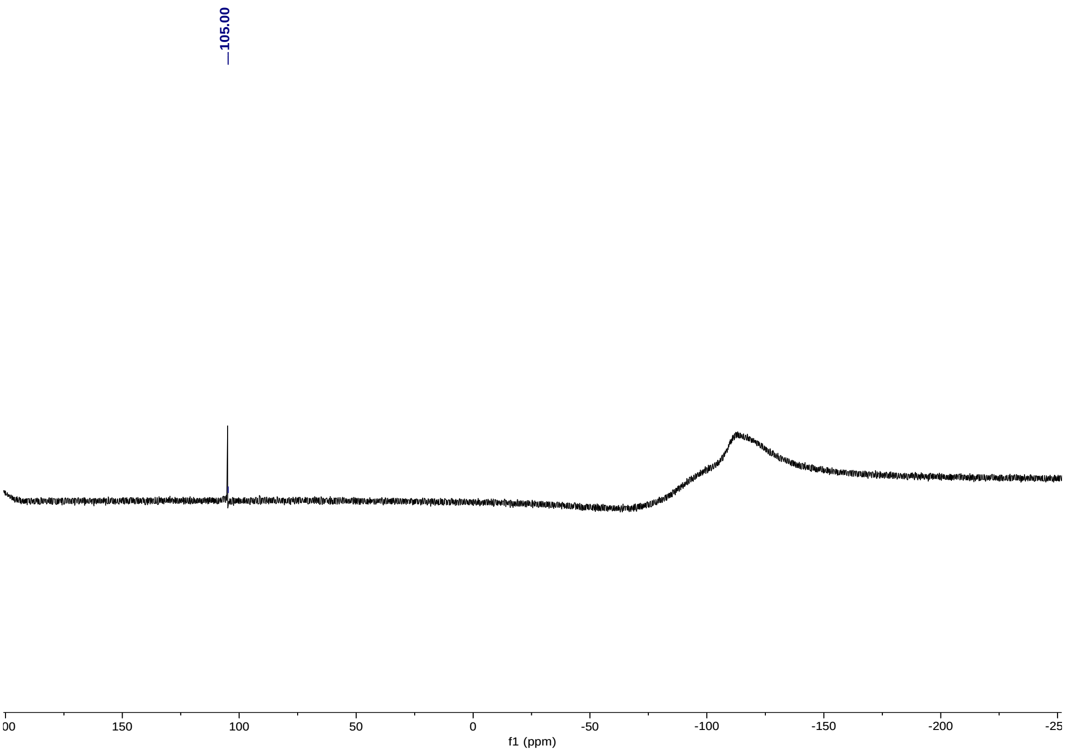

Figure S3. $^{29}Si\{^{1}H\}$ NMR spectrum of **2** in THF-d_8.

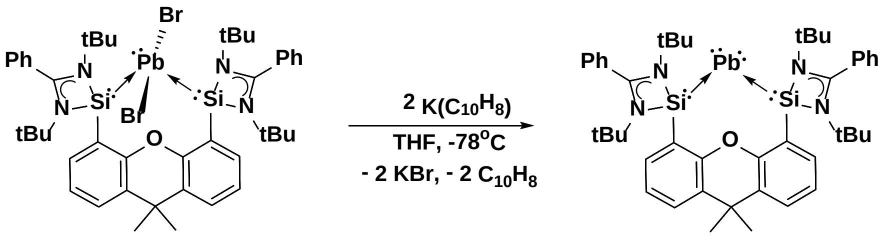

Synthesis of Compound 3. Compound **2** (55 mg, 0.05 mmol) was dissolved in THF in a 25 mL Schlenk flask, then fresh $K(C_{10}H_8)$ (0.1 mmol) was added at -78ºC under stirring, the color of the mixture changed to blue slowly. **3** was temperature sensitive-decomposing even at -50°C and decomposed to elemental lead and **1**. All attempts to isolate **3** failed due to the compound's extremely unstable nature. At -60ºC, the UV/Vis spectrum of **3** recorded in THF displays an intense absorption at 631 nm.

UV-Vis (THF, -60ºC), λ_{max}: 631 nm.

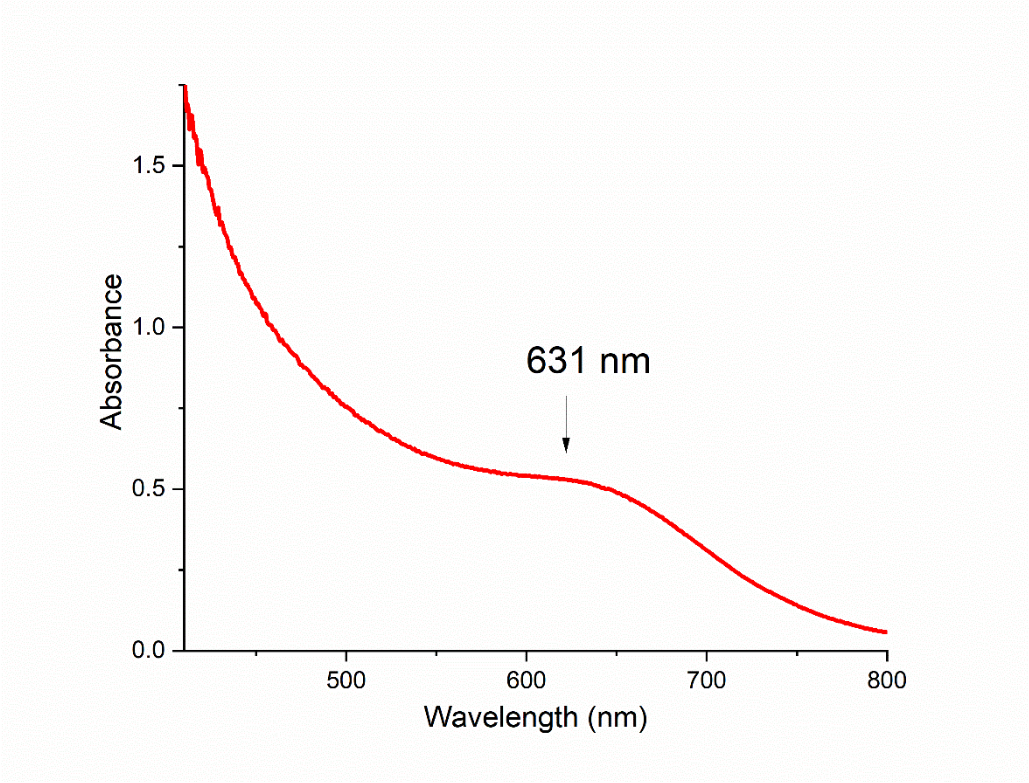

Figure S4. UV/Vis spectrum of compound **3** (-60°C, THF).

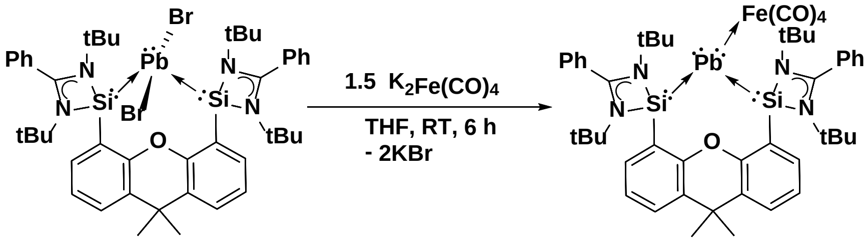

Synthesis of Compound 4. To the mixture of compound **2** (1.1 g, 1 mmol) and $K_2Fe(CO)_4$ (369 mg, 1.5 mmol) in a 100 mL Schlenk flask was added 60 mL THF at room temperature under stirring. The color of the mixture changed to red immediately. After stirring 6 h, the red mixture was filtered and the residue was washed with THF (10 mL x 2). Volatiles were removed under vacuum and the residue was washed with Et_2O (10 mL) to afford compound **4** as a red powder after dried under vacuum (825 mg, 75% isolated yield). Red block crystals suitable for X-ray single crystal diffraction analysis were obtained from a concentrated benzene solution at room temperature.

M.p. 156°C (decomp.). ^{1}H NMR (400 MHz, THF-d_8) δ = 7.81 (m, 2 H, Ar(Xant)-*H*), 7.70 – 7.67 (m, 2 H, Ar(Xant)-*H*), 7.61 (m, 4 H, Ar(Ph)-*H*), 7.58 – 7.50 (m, 6 H, Ar(Ph)-*H*), 7.24 (t, *J* = 7.5 Hz, 2 H, Ar(Xant)-*H*), 1.69 (s, 3 H, C(CH_3)$_2$), 1.58 (s, 3 H, C(CH_3)$_2$), 1.23 (s, 18 H, NC(CH_3)$_3$), 1.08 (s, 18 H, NC(CH_3)$_3$). ^{13}C{^{1}H} NMR (101 MHz, THF-d_8) δ = 220.91 (s, *C*=O), 172.22 (s, NCN), 158.24, 132.27, 132.23, 131.38, 130.52, 129.73, 129.70, 129.22, 128.82, 128.38, 124.70, 124.03 (s, Ar-*C*), 55.43 (s, N*C*(CH$_3$)$_3$), 55.07 (s, N*C*(CH$_3$)$_3$), 35.72 (s, *C*(CH$_3$)$_2$), 31.85 (s, NC(*C*H$_3$)$_3$, 31.21 (s, NC(*C*H$_3$)$_3$, 29.75 (s, C(*C*H$_3$)$_2$). ^{29}Si{^{1}H} NMR (79 MHz, THF-d_8) δ = 16.53 (s). ^{207}Pb{^{1}H} NMR (105 MHz, THF-d_8) δ = 2238.87 (s). HRMS(ESI): (m/z) calcd for $[M + H]^+$ ($C_{49}H_{59}N_4O_5Si_2PbFe$)$^+$: 1103.3134; found: 1103.3135. Elemental analysis calcd for $C_{49}H_{58}N_4O_5Si_2PbFe{\cdot}Et_2O$: C, 54.11; H, 5.83; N, 4.76 found: C, 49.65; H, 5.57; N, 4.82 [Consistently low C analysis may be due to the formation of silicon carbide]. IR (cm^{-1}): 2097(w), 1951(s), 1869(m), 1848(m), 1831(s),1472(w), 1394(s), 1366(m), 1278(w), 1206(w),1118(w), 1088(w), 1023(w), 712(w), 706(w), 631(w), 618(s). UV-Vis (THF)： λ_{max} (ε_{max}/(M^{-1} cm^{-1}): 431 nm (4848).

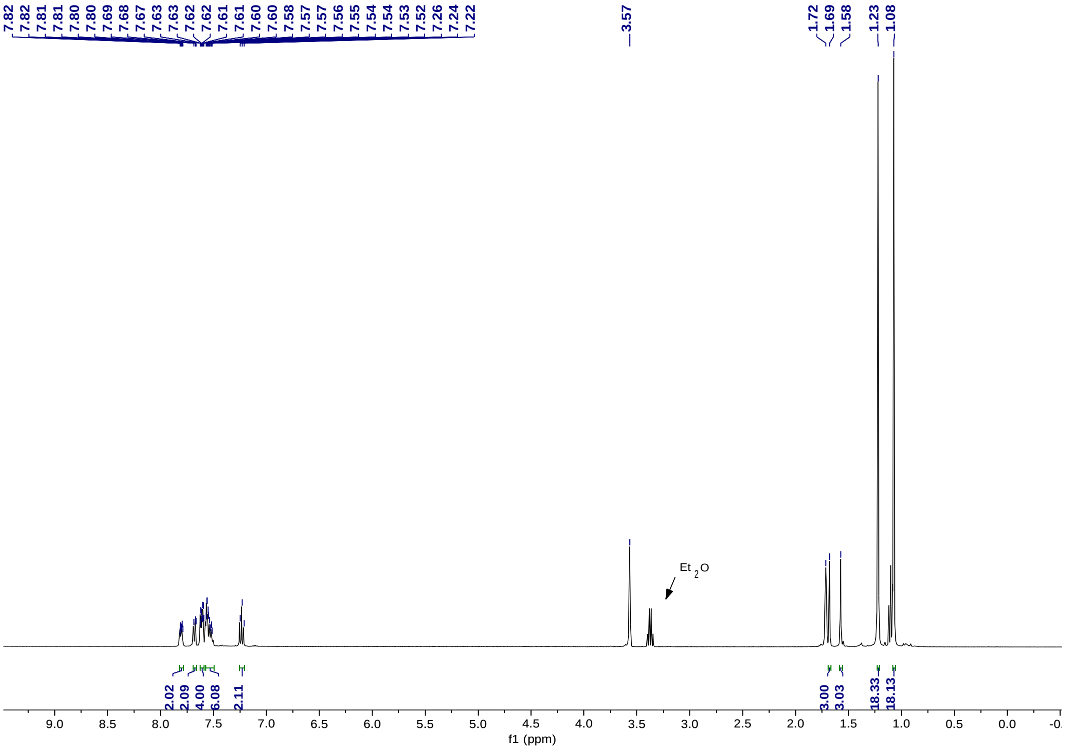

Figure S5. ^{1}H NMR spectrum of **4** in THF-d_8.

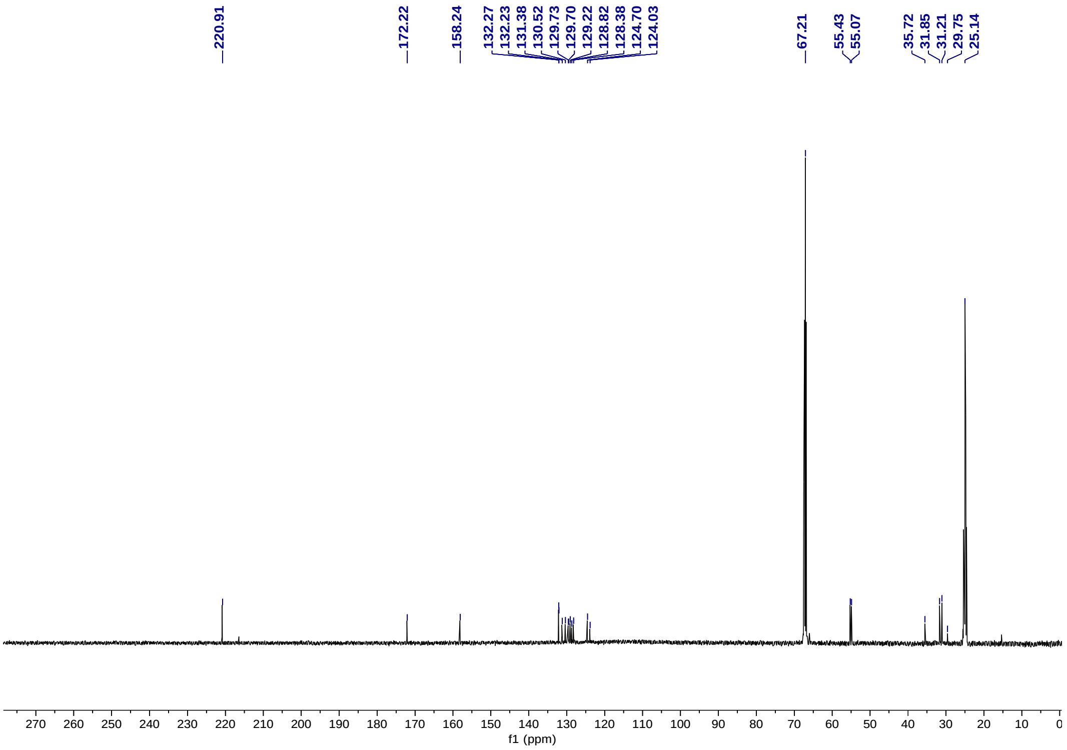

Figure S6. $^{13}C\{^{1}H\}$ NMR spectrum of **4** in THF-*d*₈.

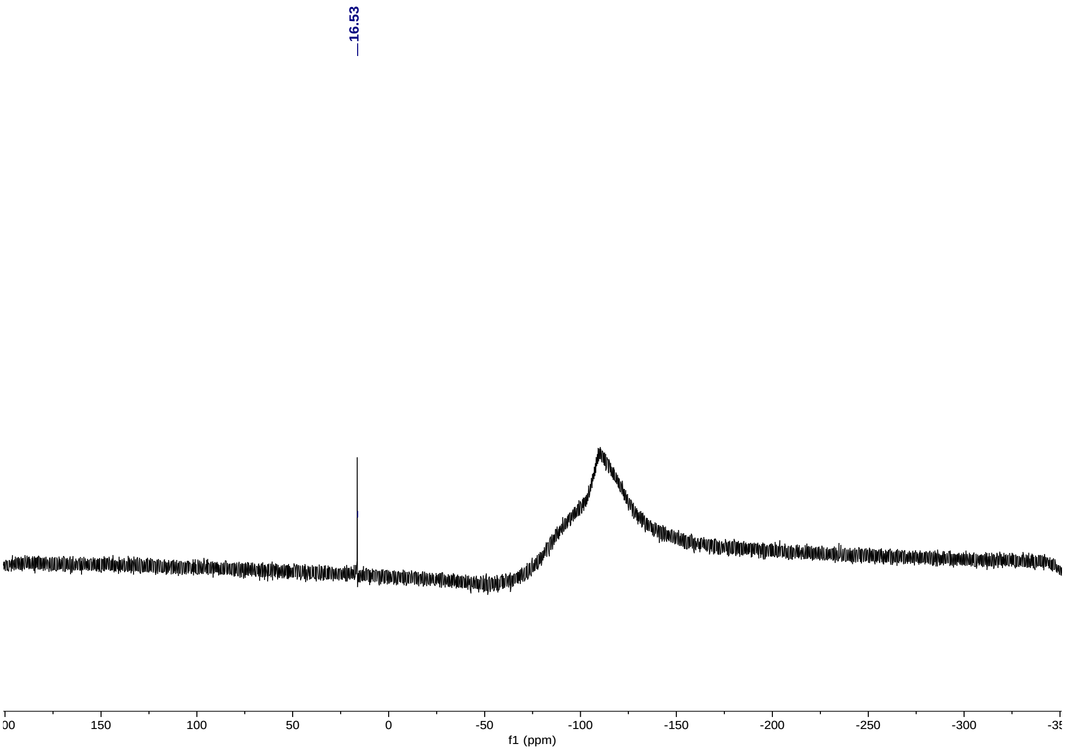

Figure S7. $^{29}Si\{^{1}H\}$ spectrum of **4** in THF-*d*₈.

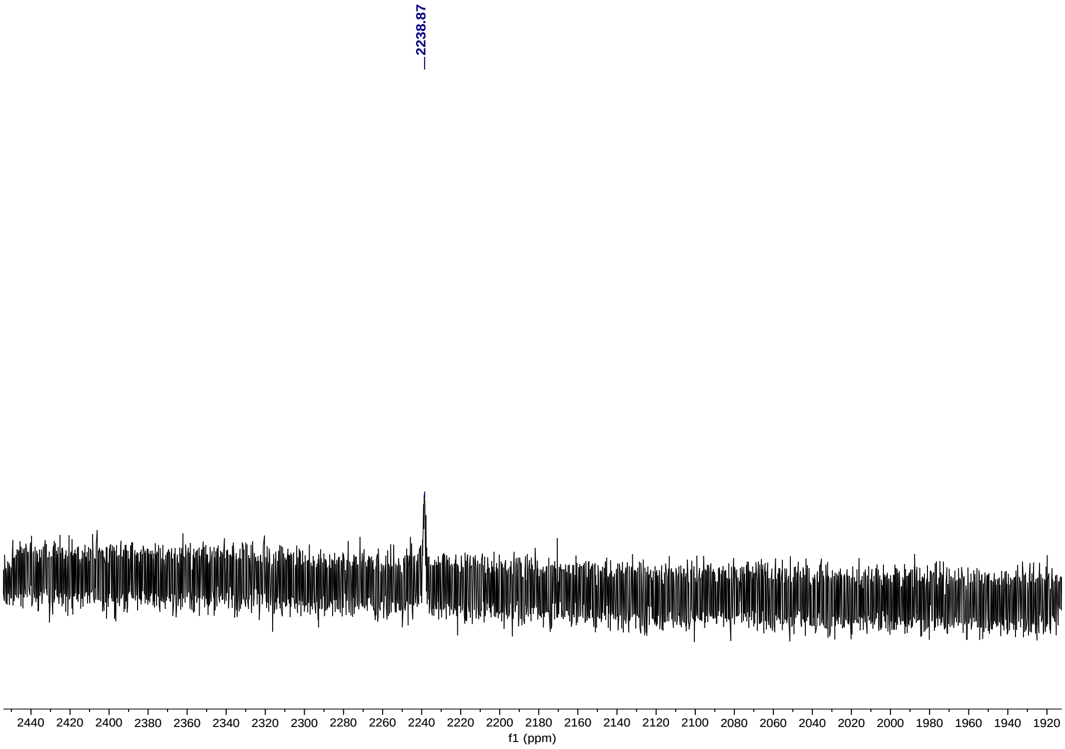

Figure S8. $^{207}Pb\{^{1}H\}$ spectrum of **4** in THF-*d*$_8$.

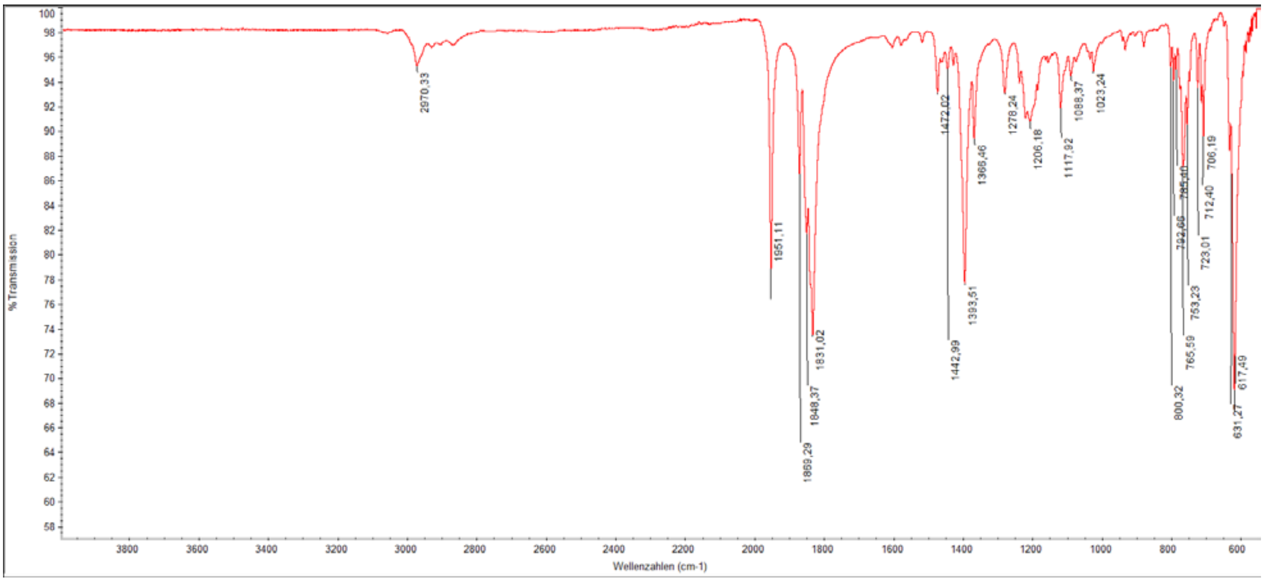

Figure S9. IR spectrum of **4**.

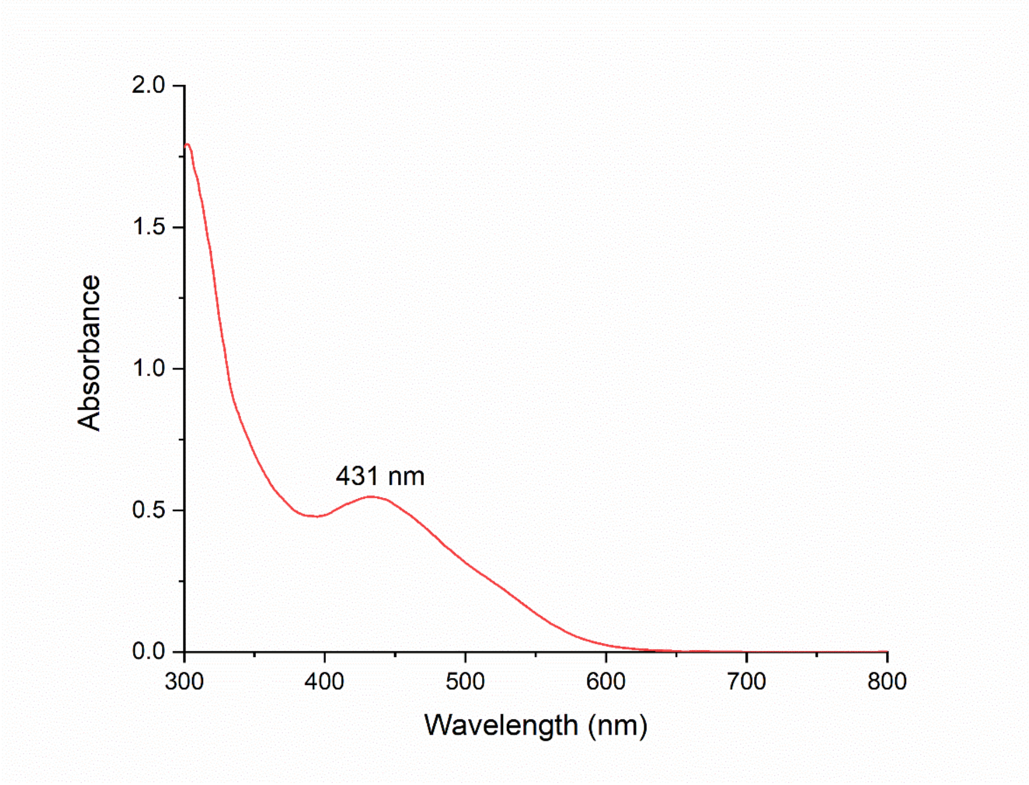

Figure S10. UV/Vis spectrum of **4** (RT, THF, 1.13×10^{-4} M).

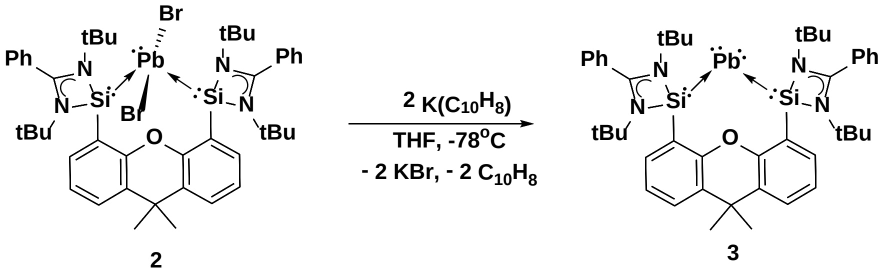

Reduction of Compound 4. Compound **4** (110 mg, 0.1 mmol) was dissolved in THF in a 25 mL Schlenk flask, then fresh K($C_{10}H_8$) (0.2 mmol) was added at -78°C under stirring, the color of the mixture changed to blue slowly. All attempts to isolate **3** failed again due to the compound's extremely unstable nature. The UV/Vis spectrum of THF soultion displays an intense absorption at 631 nm at -60 ^{o}C.

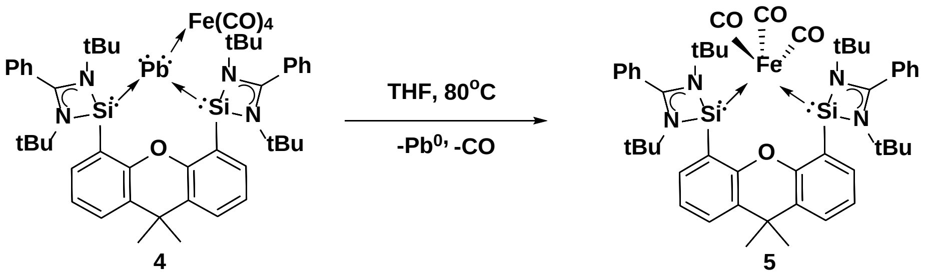

Synthesis of Compound 5. The THF solution of compound **4** (110 mg, 0.1 mmol) in a 25 mL Schlenk flask was heated at 80°C under stirring. The color of the mixture changed from red to yellow. After stirring 2 h, the deep yellow mixture was filtered, volatiles were removed under vacuum and the residue was washed with hexane (5 mL) to afford compound **5** as a yellow powder after dried under vacuum (52 mg, 60% isolated yield). Yellow block crystals suitable for X-ray single crystal diffraction analysis were obtained from a concentrated benzene solution at room temperature.

M.p. 263°C (decomp.). ^{1}H NMR (200 MHz, C_6D_6) δ = 7.90 (dd, *J* = 7.0, 1.8 Hz, 2 H, Ar(Xant)-*H*), 7.44 – 7.38 (m, 2 H, Ar(Xant)-*H*), 7.34 (d, *J* = 7.2 Hz, 2 H, Ar(Xant)-*H*), 7.06 – 7.00 (m, 4 H, Ar(Ph)-*H*), 6.95 – 6.85 (m, 6 H, Ar(Ph)-*H*), 1.55 (s, 6 H, C(CH_3)$_2$), 1.37 (s, 36 H, NC(CH_3)$_3$). ^{13}C{^{1}H} NMR (50 MHz, C_6D_6) δ = 229.03 (s, *C*=O), 170.78 (s, N*C*N), 160.96, 134.89, 132.90, 130.66, 130.07, 129.90, 129.76, 128.35, 127.86, 127.36, 125.96, 123.15 (s, Ar-*C*), 54.73 (s, N*C*(CH_3)$_3$), 36.52 (s, *C*(CH_3)$_2$), 31.27 (s, NC(*C*H_3)$_3$), 27.76(s, C(*C*H_3)$_2$). ^{29}Si{^{1}H} NMR (79 MHz, C_6D_6) δ = 94.68 (s). HRMS(ESI): (m/z) calcd for [M] ($C_{48}H_{58}N_4O_4Si_2Fe$): 866.3341; found: 866.3337. Elemental analysis calcd for $C_{48}H_{58}N_4O_4Si_2Fe$: C, 66.49; H, 6.74; N, 6.46 found: C, 65.55; H, 6.87; N, 6.24. IR (cm-1):1936(m), 1876(m), 1855(s), 1416(m), 1388(s), 1213(m), 758(s), 708(m), 646(s), 635(s), 626(s).

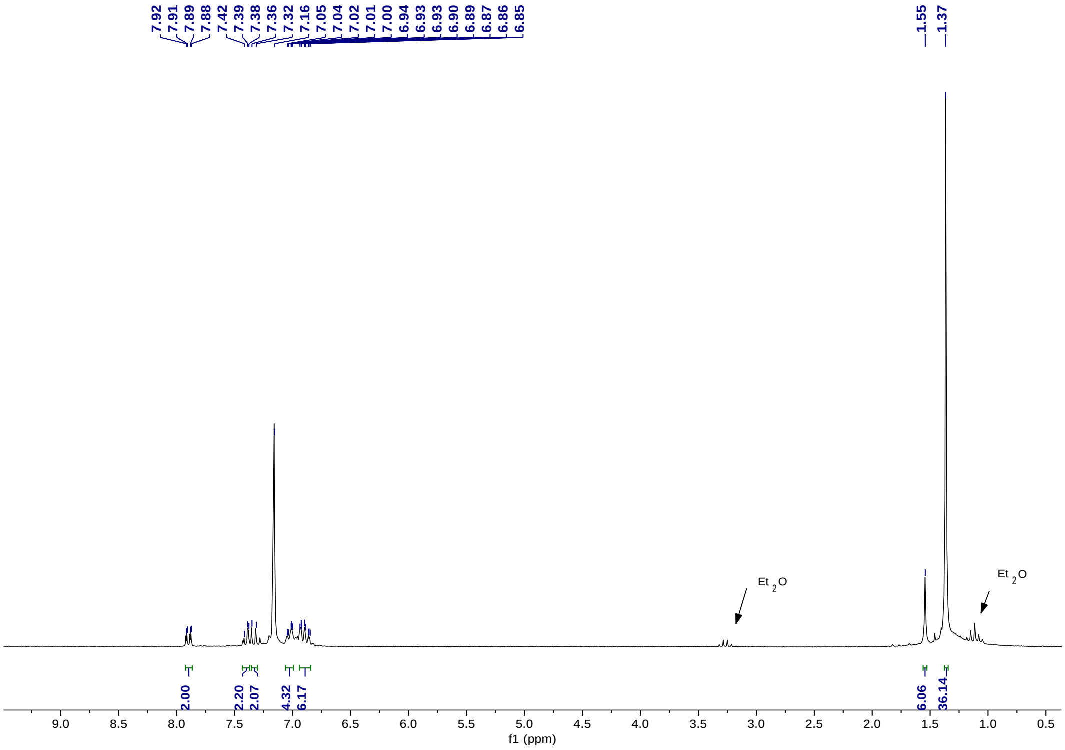

Figure S11. ^{1}H NMR spectrum of **5** in C_6D_6.

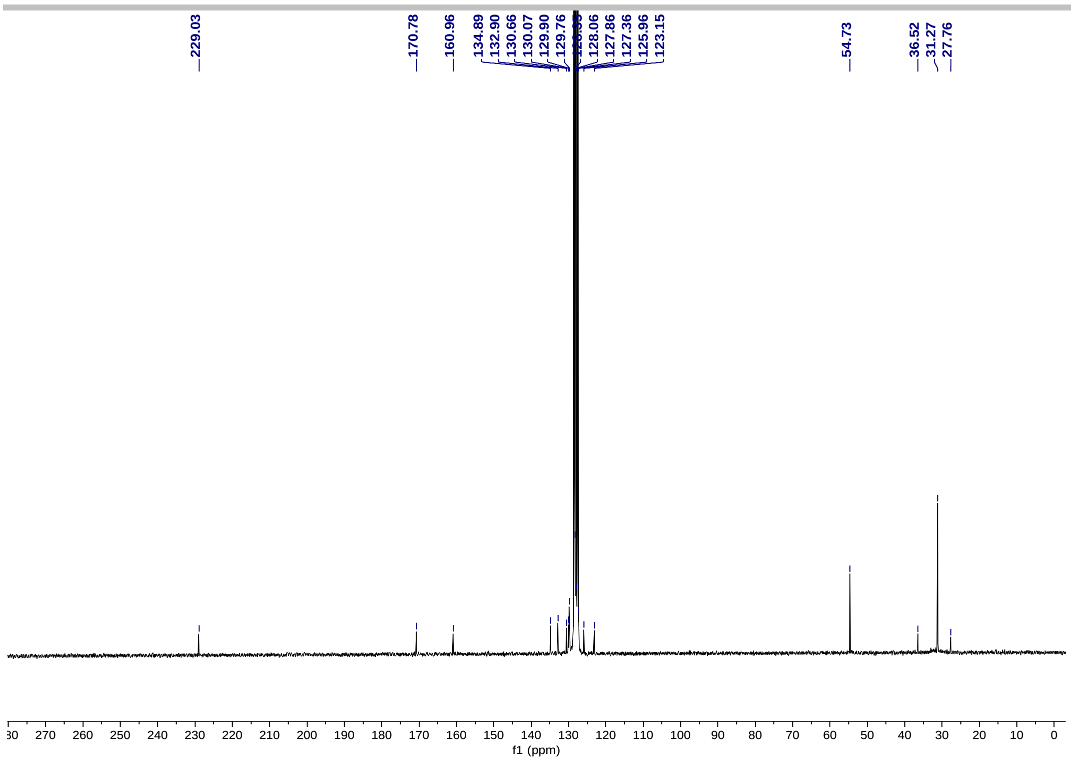

Figure S12. $^{13}C\{^{1}H\}$ NMR spectrum of **5** in C_6D_6.

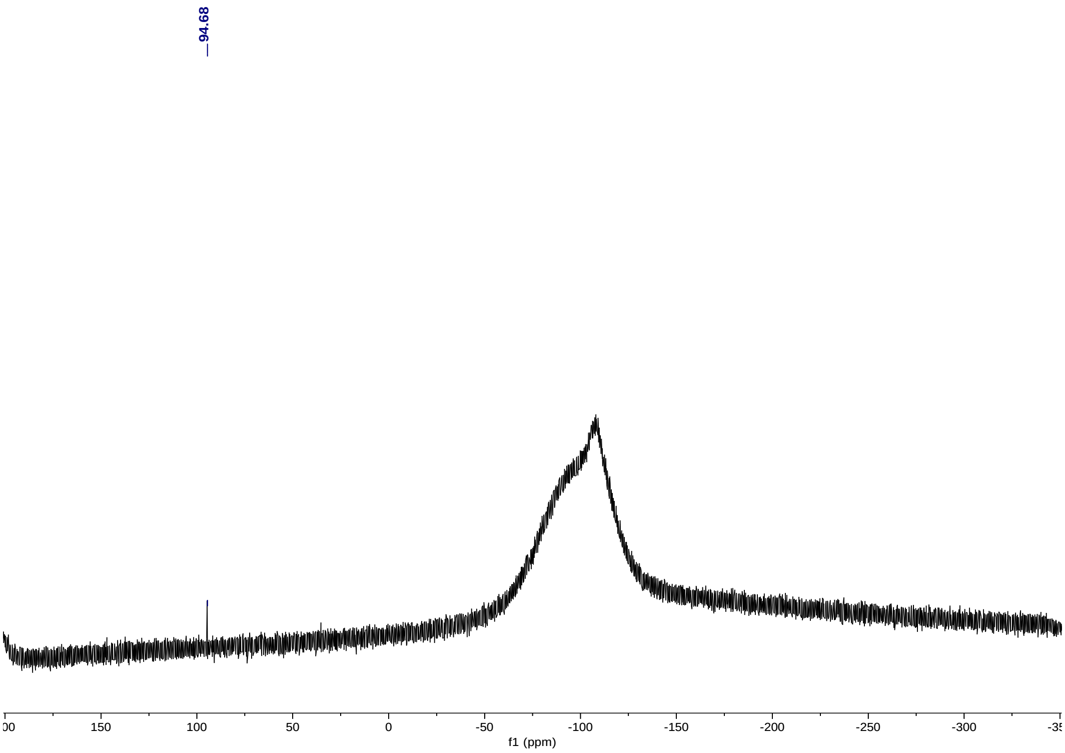

Figure S13. $^{29}Si\{^{1}H\}$ spectrum of **5** in C_6D_6.

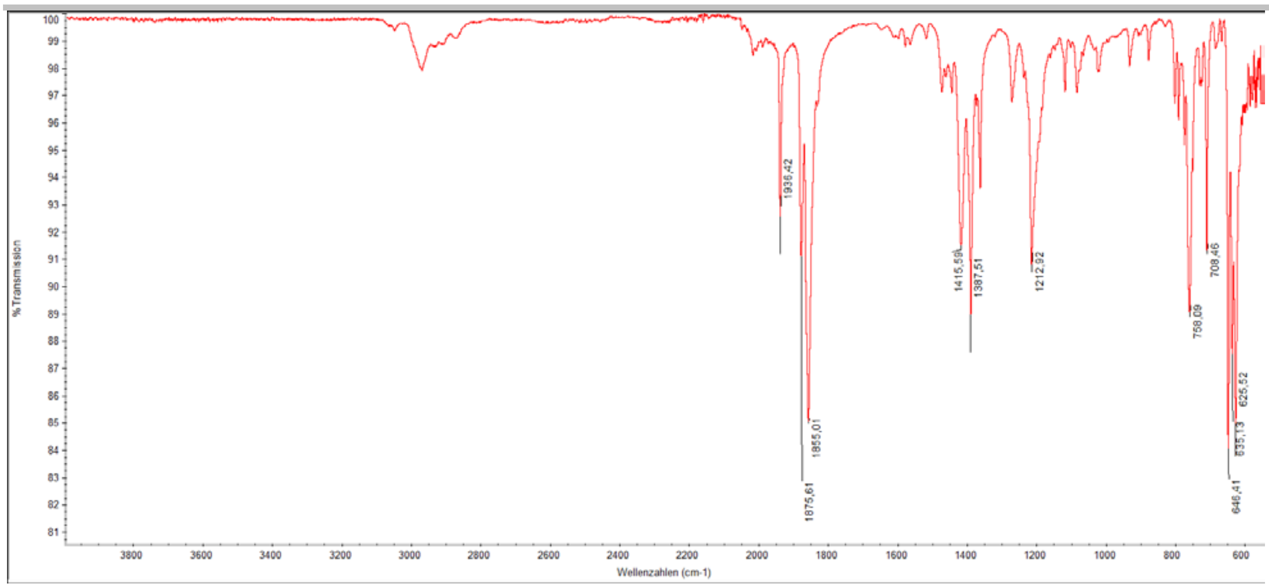

Figure S14. IR spectrum of **5**.

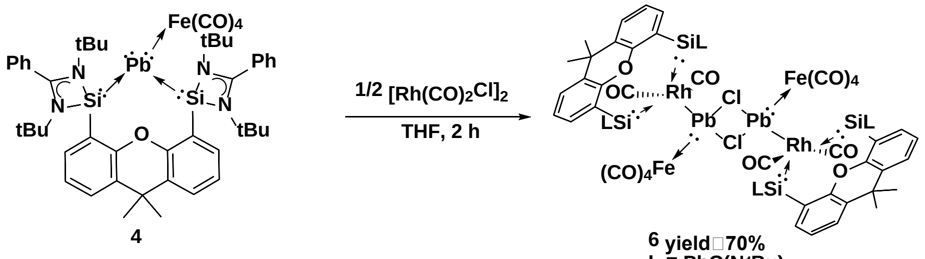

4

6 yield 70%
L = PhC(NtBu)2

Reaction of 4 with [Rh(CO)$_2$Cl]$_2$. To the mixture of **4** (220 mg, 0.2 mmol) and [Rh(CO)$_2$Cl]$_2$ (38.8 mg, 0.1 mmol) in a 25 mL Schlenk flask was added 10 mL THF at room temperature under stirring. The color of the mixture changed to dark brown immediately. After stirring for 2 h, Volatiles were removed under vacuum and the residue was washed with Et$_2$O (5 mL) to afford compound **6** as a dark brown powder after dried under vacuum (208 mg, 70% isolated yield). Dark brown block crystals suitable for X-ray diffraction analysis were obtained from a concentrated benzene solution at room temperature.

M.p. 207°C (decomp.). ^{1}H NMR (400 MHz, THF-d_8) δ = 7.94 – 7.81 (m, 2 H, Ar(Xant)-*H*), 7.78 (d, *J* = 7.4 Hz, 2 H, Ar(Xant)-*H*), 7.72 (d, *J* = 7.4 Hz, 2 H, Ar(Xant)-*H*), 7.57-7.66 (m, 8 H, Ar(Ph)-*H*), 7.28 (t, *J* = 7.0 Hz, 2H, Ar(Ph)-*H*), 1.60 (s, 6 H, C(C*H*$_3$)$_2$), 1.25 (s, 36 H, NC(C*H*$_3$)$_3$). ^{13}C{^{1}H} NMR (50 MHz, THF-d_8) δ = 215.98, 211.26 (s, *C*=O), 173.40 (s, N*C*N), 158.90, 134.42, 132.05, 131.70, 130.69, 130.55, 129.44, 129.13, 128.67, 128.18, 125.81, 124.35 (s, Ar-*C*), 55.72 (s, N*C*(CH$_3$)$_3$), 36.17 (s, *C*(CH$_3$)$_2$), 31.54 (s, NC(*C*H$_3$)$_3$), 28.93 (s, C(*C*H$_3$)$_2$). ^{29}Si{^{1}H} NMR (79 MHz, THF-d_8) δ = 77.45 (d, $J_{Rh\text{-}Si}$ = 63.99 Hz). Elemental analysis calcd for $C_{102}H_{116}N_8O_{14}Si_4Fe_2Rh_2Pb_2Cl_2{\cdot}6C_6H_6$ (crystal): C, 54.13; H, 5.00; N, 3.66 found: C, 41.17; H, 4.71; N, 3.80 [Consistently low C analysis may be due to the formation of silicon carbide]. IR (cm^{-1}): 2972(w), 1988(s), 1970(m), 1917(m), 1902(s), 1848(m), 1578(w), 1517(w), 1391(s), 1365(m), 1201(m), 1118(w), 1022(w), 800(w), 764(m), 707(m), 609(s).

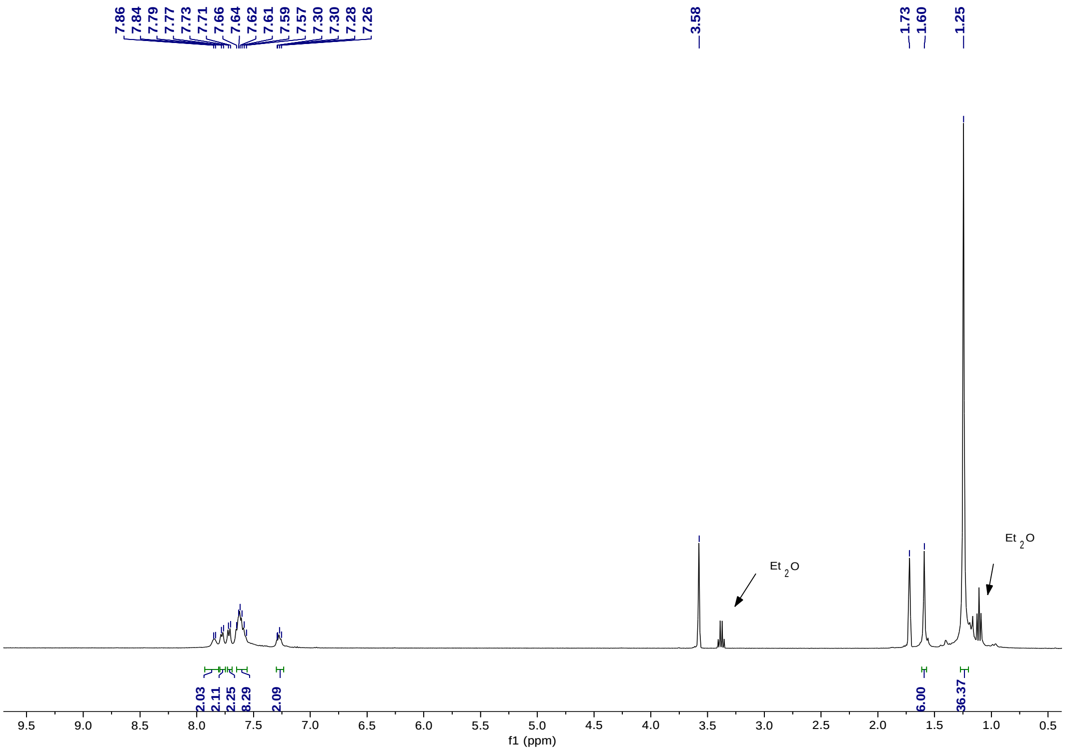

Figure S15. ^{1}H NMR spectrum of **6** in THF-d_8.

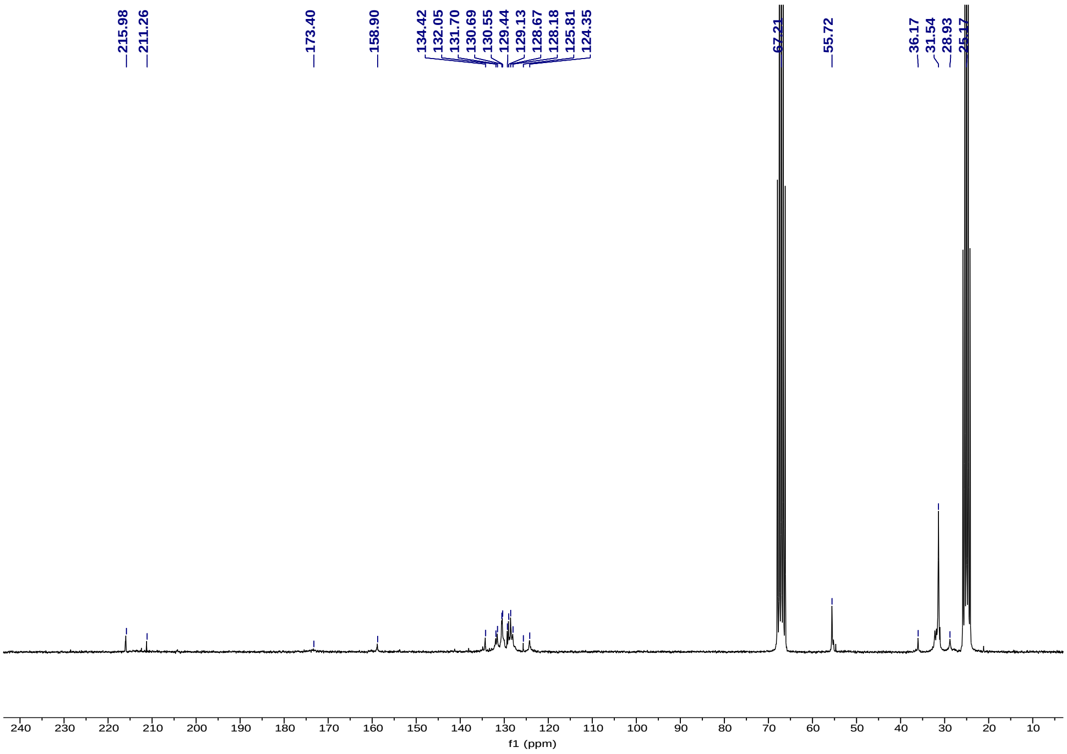

Figure S16. $^{13}C\{^{1}H\}$ NMR spectrum of **6** in THF-d_8.

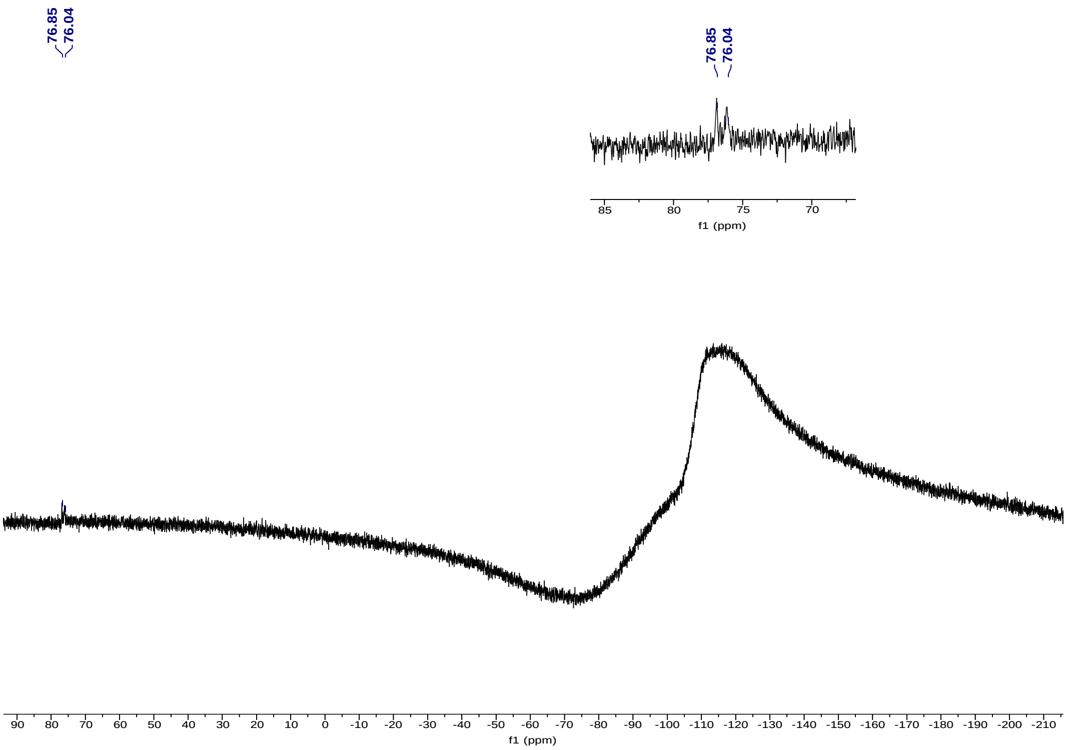

Figure S17. $^{29}Si\{^{1}H\}$ NMR spectrum of **6** in THF-d_8.

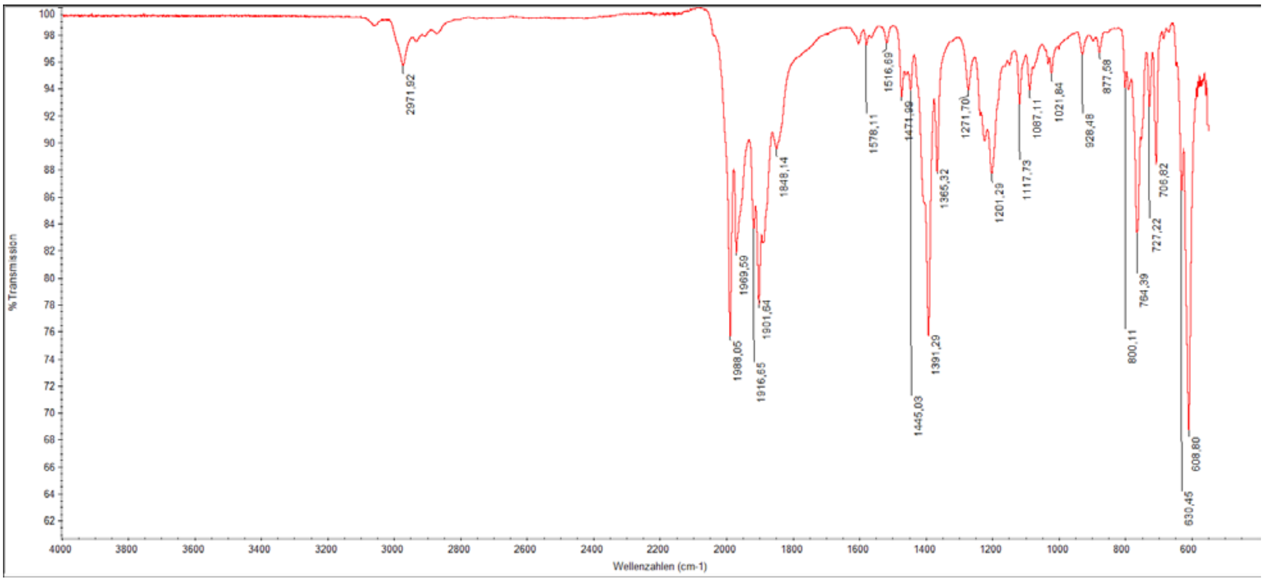

Figure S18. IR spectrum of **6**.

A4. X-ray Crystallographic Data

Table S1. Crystallographic data and structure refinement for compound **2 [3·THF]**

Empirical formula	C57 H83 Br2 N4 O4 Pb Si2	
Formula weight	1309.47	
Temperature	150.00(10) K	
Wavelength	1.54184 Å	
Crystal system	Monoclinic	
Space group	C 1 2/c 1	
Unit cell dimensions	a = 25.6559(3) Å	a= 90°.
	b = 19.6872(2) Å	b= 99.5930(10)°.
	c = 23.9043(2) Å	g = 90°.
Volume	11905.1(2) $Å^3$	
Z	8	
Density (calculated)	1.461 Mg/m^3	
Absorption coefficient	7.815 mm^{-1}	
F(000)	5312	
Crystal size	0.05 x 0.02 x 0.01 mm^3	
Theta range for data collection	2.844 to 67.495°.	
Index ranges	-29<=h<=30, -23<=k<=17, -28<=l<=28	
Reflections collected	43239	
Independent reflections	10740 [R(int) = 0.0420]	
Completeness to theta = 67.495°	100.0 %	
Absorption correction	Semi-empirical from equivalents	
Max. and min. transmission	1.00000 and 0.01507	
Refinement method	Full-matrix least-squares on F^2	
Data / restraints / parameters	10740 / 0 / 510	
Goodness-of-fit on F^2	1.040	
Final R indices [I>2sigma(I)]	R1 = 0.0341, wR2 = 0.0891	
R indices (all data)	R1 = 0.0398, wR2 = 0.0945	
Extinction coefficient	n/a	
Largest diff. peak and hole	1.555 and -1.217 e.$Å^{-3}$	

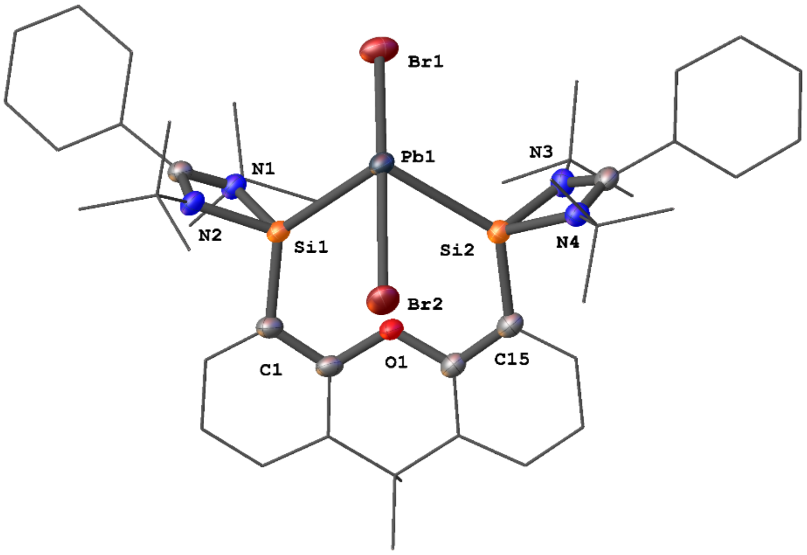

Figure **S19**. Molecular structure of compound **2 [3·THF]**. Thermal ellipsoids are drawn at the 50% probability level. H atoms are omitted for clarity. The strongly disordered THF molecules are treated using Solvent Masking in Olex2.

Table S2. Selected interatomic distances and angles of compound **2**.

Interatomic distances(Å)		Angles(°)	
Pb(1)-Br(1)	2.9693(4)	Br(1)-Pb(1)-Br(2)	166.547(14)
Pb(1)-Br(2)	2.9886(4)	Si(1)-Pb(1)-Br(1)	88.76(2)
Pb(1)-Si(1)	2.7594(9)	Si(1)-Pb(1)-Br(2)	81.55(2)
Pb(1)-Si(2)	2.7750(10)	Si(1)-Pb(1)-Si(2)	99.98(3)
Si(1)-N(1)	1.833(3)	Si(2)-Pb(1)-Br(1)	96.51(2)
Si(1)-N(2)	1.829(3)	Si(2)-Pb(1)-Br(2)	76.18(2)
Si(1)-C(31)	2.274(4)	N(1)-Si(1)-Pb(1)	120.09(10)
Si(1)-C(1)	1.882(4)	N(1)-Si(1)-C(1)	104.11(15)
Si(2)-N(3)	1.829(3)	N(2)-Si(1)-Pb(1)	109.90(10)
Si(2)-N(4)	1.852(3)	N(2)-Si(1)-N(1)	71.26(13)
		N(2)-Si(1)-C(1)	106.19(16)
		C(1)-Si(1)-Pb(1)	129.55(12)
		N(3)-Si(2)-Pb(1)	119.58(11)
		N(3)-Si(2)-N(4)	71.14(14)

Table S3. Crystallographic data and structure refinement for compound **4**

Empirical formula	C55 H64 Fe N4 O5 Pb Si2	
Formula weight	1180.32	
Temperature	150(10) K	
Wavelength	1.54184 Å	
Crystal system	Monoclinic	
Space group	P 1 21/c 1	
Unit cell dimensions	a = 10.70110(10) Å	a= 90°.
	b = 22.6825(2) Å	b= 93.2930(10)°.
	c = 22.2419(2) Å	g = 90°.
Volume	5389.81(8) Å^3	
Z	4	
Density (calculated)	1.455 Mg/m^3	
Absorption coefficient	8.975 mm^{-1}	
F(000)	2392	
Crystal size	0.08 x 0.05 x 0.04 mm^3	
Theta range for data collection	2.785 to 67.494°.	
Index ranges	-12<=h<=12, -27<=k<=26, -26<=l<=22	
Reflections collected	21366	
Independent reflections	9694 [R(int) = 0.0264]	
Completeness to theta = 67.494°	99.9 %	
Absorption correction	Semi-empirical from equivalents	
Max. and min. transmission	1.00000 and 0.21847	
Refinement method	Full-matrix least-squares on F^2	
Data / restraints / parameters	9694 / 0 / 637	
Goodness-of-fit on F^2	1.085	
Final R indices [I>2sigma(I)]	R1 = 0.0271, wR2 = 0.0678	
R indices (all data)	R1 = 0.0299, wR2 = 0.0717	
Extinction coefficient	n/a	
Largest diff. peak and hole	1.157 and -1.301 e.Å^{-3}	

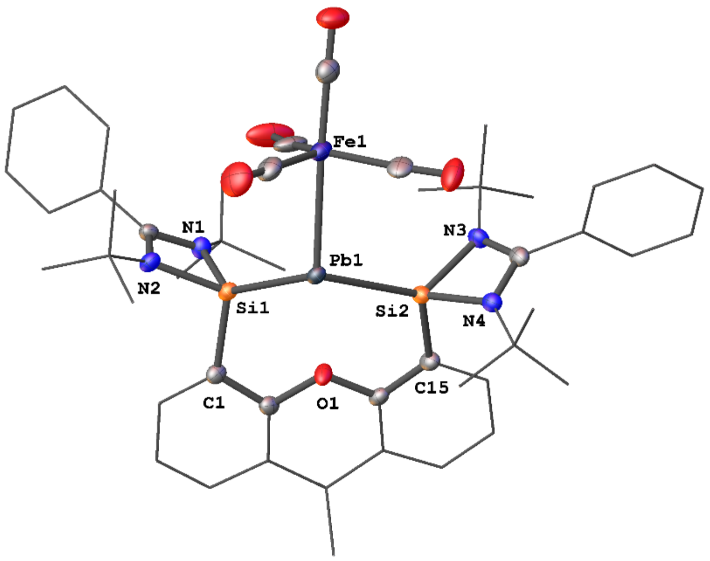

Figure **S20**. Molecular structure of compound **4**. Thermal ellipsoids are drawn at the 50% probability level. H atoms and solvent C_6H_6 molecules are omitted for clarity. The lead atom is disordered over two positions with an approximate occupancy ratio of 0.98 : 0.02.

Table S4. Selected interatomic distances and angles of compound **4**.

Interatomic distances(Å)		Angles(°)	
Pb(1)-Fe(1)	2.7367(5)	Fe(1)-Pb(1)-Si(1)	106.211(19)
Pb(1)-Si(1)	2.7948(8)	Fe(1)-Pb(1)-Si(2)	102.770(18)
Pb(1)-Si(2)	2.7914(7)	Si(2)-Pb(1)-Si(1)	97.55(2)
Fe(1)-Pb(1A)	2.385(5)	N(1)-Si(1)-Pb(1)	136.32(8)
Si(1)-N(1)	1.840(3)	N(1)-Si(1)-Pb(1A)	92.14(14)
Si(1)-N(2)	1.832(3)	N(2)-Si(1)-Pb(1)	117.45(8)
Si(1)-C(1)	1.893(3)	N(2)-Si(1)-Pb(1A)	113.90(14)
Si(1)-Pb(1A)	2.643(6)	C(1)-Si(1)-Pb(1)	115.45(9)
Si(2)-N(4)	1.842(3)	C(1)-Si(1)-Pb(1A)	141.18(15)
Si(2)-N(3)	1.835(2)	C(15)-Si(2)-Pb(1)	123.87(9)
Si(2)-C(15)	1.890(3)	C(15)-Si(2)-Pb(1A)	124.77(15)
Si(2)-Pb(1A)	2.906(5)	N(2)-Si(1)-N(1)	71.11(11)
		N(3)-Si(2)-N(4)	70.58(11)

Table S5. Crystallographic data and structure refinement for compound **5 [3·C_6H_6]**

Empirical formula	C48 H58 Fe N4 O4 Si2	
Formula weight	867.01	
Temperature	150.00(10) K	
Wavelength	1.54184 Å	
Crystal system	Monoclinic	
Space group	P 1 21/n 1	
Unit cell dimensions	a = 11.7648(2) Å	a= 90°.
	b = 25.4411(4) Å	b= 103.813(2)°.
	c = 21.1799(3) Å	g = 90°.
Volume	6156.01(17) $Å^3$	
Z	4	
Density (calculated)	0.935 Mg/m^3	
Absorption coefficient	2.612 mm^{-1}	
F(000)	1840	
Crystal size	0.06 x 0.05 x 0.04 mm^3	
Theta range for data collection	2.763 to 67.495°.	
Index ranges	-13<=h<=14, -30<=k<=30, -25<=l<=23	
Reflections collected	24371	
Independent reflections	11045 [R(int) = 0.0317]	
Completeness to theta = 67.495°	99.6 %	
Absorption correction	Semi-empirical from equivalents	
Max. and min. transmission	1.00000 and 0.20757	
Refinement method	Full-matrix least-squares on F^2	
Data / restraints / parameters	11045 / 0 / 546	
Goodness-of-fit on F^2	1.052	
Final R indices [I>2sigma(I)]	R1 = 0.0506, wR2 = 0.1573	
R indices (all data)	R1 = 0.0627, wR2 = 0.1694	
Extinction coefficient	n/a	
Largest diff. peak and hole	0.386 and -0.374 e.$Å^{-3}$	

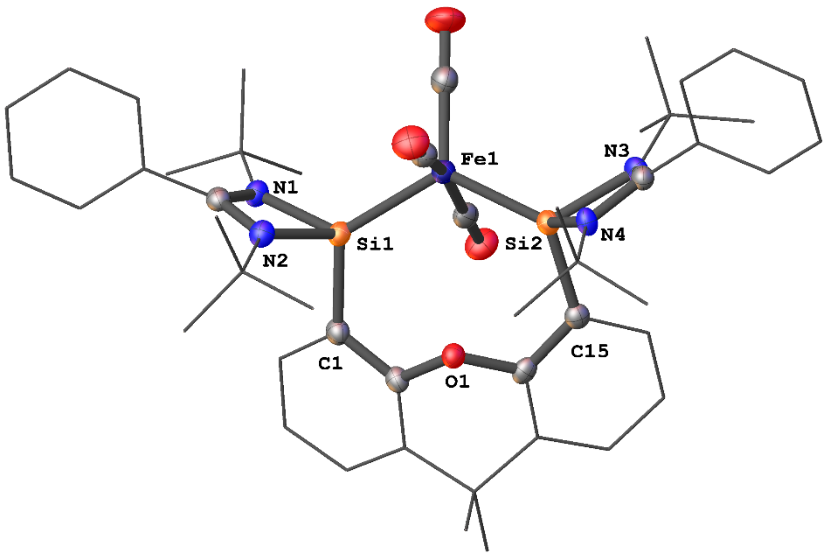

Figure **S21**. Molecular structure of compound **5 [3·C_6H_6]**. Thermal ellipsoids are drawn at 50% probability level. Hydrogen atoms are omitted for clarity. The strongly disordered C_6H_6 molecules are treated using Solvent Masking in Olex2.

Table S6. Selected interatomic distances and angles of compound **5**.

Interatomic distances(Å)		Angles(°)	
Fe(01)-Si(2)	2.2090(8)	Si(1)-Fe(01)-Si(2)	123.15(3)
Fe(01)-Si(1)	2.2076(8)	Fe(01)-Si(2)-C(16)	131.12(7)
Fe(01)-C(48)	1.753(3)	N(4)-Si(2)-Fe(01)	126.19(8)
Fe(01)-C(47)	1.757(3)	N(4)-Si(2)-N(3)	70.53(10)
Fe(01)-C(46)	1.757(3)	N(4)-Si(2)-C(15)	103.81(10)
Si(2)-N(4)	1.855(2)	N(4)-Si(2)-C(16)	35.39(9)
Si(2)-N(3)	1.864(2)	N(3)-Si(2)-Fe(01)	121.96(7)
Si(2)-C(15)	1.908(3)	N(3)-Si(2)-C(15)	102.54(11)
Si(2)-C(16)	2.316(3)	N(3)-Si(2)-C(16)	35.25(9)
Si(1)-N(2)	1.853(2)	C(15)-Si(2)-Fe(01)	120.30(8)
		C(15)-Si(2)-C(16)	108.38(10)
		Fe(01)-Si(1)-C(31)	131.81(7)
		N(2)-Si(1)-Fe(01)	126.97(8)

Table S7. Crystallographic data and structure refinement for compound **6**

Empirical formula	C69 H76 Cl Fe N4 O7 Pb Rh Si2	
Formula weight	1530.91	
Temperature	104(3) K	
Wavelength	1.54184 Å	
Crystal system	Monoclinic	
Space group	P 1 21/n 1	
Unit cell dimensions	a = 21.32995(17) Å	a= 90°.
	b = 15.40461(10) Å	b= 103.8956(8)°.
	c = 21.56705(19) Å	g = 90°.
Volume	6879.10(10) $Å^3$	
Z	4	
Density (calculated)	1.478 Mg/m^3	
Absorption coefficient	9.382 mm^{-1}	
F(000)	3088	
Crystal size	0.07 x 0.03 x 0.01 mm^3	
Theta range for data collection	2.616 to 67.500°.	
Index ranges	-25<=h<=24, -18<=k<=18, -25<=l<=25	
Reflections collected	49037	
Independent reflections	12406 [R(int) = 0.0329]	
Completeness to theta = 67.500°	100.0 %	
Absorption correction	Semi-empirical from equivalents	
Max. and min. transmission	1.00000 and 0.21713	
Refinement method	Full-matrix least-squares on F^2	
Data / restraints / parameters	12406 / 0 / 789	
Goodness-of-fit on F^2	1.108	
Final R indices [I>2sigma(I)]	R1 = 0.0285, wR2 = 0.0729	
R indices (all data)	R1 = 0.0301, wR2 = 0.0739	
Extinction coefficient	n/a	
Largest diff. peak and hole	1.040 and -2.160 e.$Å^{-3}$	

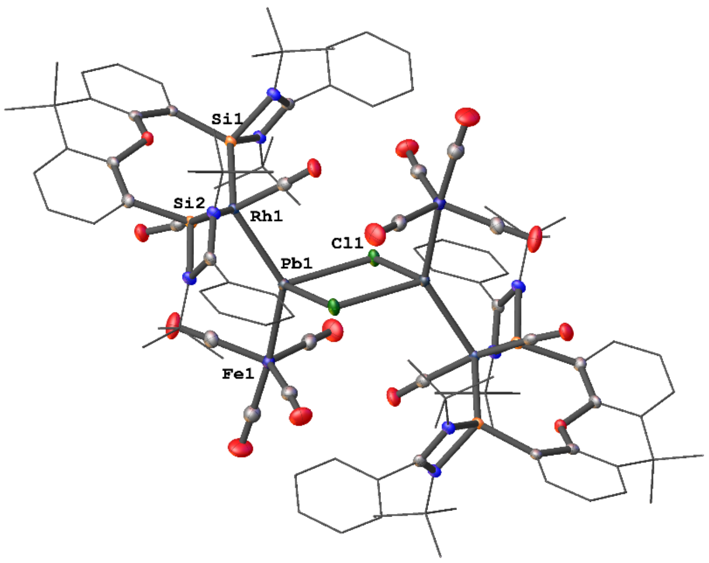

Figure **S22**. Molecular structure of compound **6**. Thermal ellipsoids are drawn at 50% probability level. H atoms and solvent C_6H_6 molecules are omitted for clarity.

Table S8. Selected interatomic distances and angles of compound **6**.

Interatomic distances(Å)		Angles(°)	
Pb(1)-Rh(1)	2.6943(2)	Rh(1)-Pb(1)-Cl(1)	109.936(16)
Pb(1)-Fe(1)	2.6052(5)	Rh(1)-Pb(1)-Cl(1)#1	103.306(16)
Pb(1)-Cl(1)#1	2.8182(7)	Fe(1)-Pb(1)-Rh(1)	136.561(12)
Pb(1)-Cl(1)	2.7731(7)	Fe(1)-Pb(1)-Cl(1)	105.690(19)
Rh(1)-Si(2)	2.3334(7)	Fe(1)-Pb(1)-Cl(1)#1	108.000(19)
Rh(1)-Si(1)	2.3358(7)	Cl(1)-Pb(1)-Cl(1)#1	77.54(2)
Rh(1)-C(46)	1.895(3)	Si(2)-Rh(1)-Pb(1)	112.19(2)
Rh(1)-C(47)	1.887(3)	Si(2)-Rh(1)-Si(1)	125.65(3)
Cl(1)-Pb(1)#1	2.8182(7)	Si(1)-Rh(1)-Pb(1)	121.97(2)
Si(2)-C(15)	1.892(3)	Pb(1)-Cl(1)-Pb(1)#1	102.46(2)
Si(1)-C(1)	1.898(3)	C(15)-Si(2)-Rh(1)	118.32(10)
Pb(1)-Rh(1)	2.6943(2)	C(1)-Si(1)-Rh(1)	118.89(9)

B. Theoretical Calculations

The geometry optimizations and the harmonic vibrational frequency calculations of complexes **3** and **4** were performed at the BP86-D3(BJ)/def2-TZVP level.[4–7] The partial atomic charges were computed using the NBO6 program[8] at the same level. TDDFT calculations were performed at the CAM-B3LYP-D3(BJ)/def2-TZVP//BP86-D3(BJ)/def2-TZVP level[9] to simulate UV-Vis spectra. All these calculations were performed using Gaussian 16 program.[10]

The bonding situations were further analysed by means of an energy decomposition analysis (EDA)[11] together with the natural orbitals for chemical valence (NOCV)[12,13] method by using the ADF 2018.105 program package.[14,15] The EDA-NOCV calculations[16,17] were carried out at the BP86-D3(BJ)/TZ2P-ZORA//BP86-D3(BJ)/def2-TZVP level.[18] In this analysis, the intrinsic interaction energy (ΔE_{int}) between two fragments can be divided into three energy components as follows:

$$\Delta E_{\text{int}} = \Delta E_{\text{elstat}} + \Delta E_{\text{Pauli}} + \Delta E_{\text{orb}} + \Delta E_{\text{disp}} \qquad (1).$$

The electrostatic ΔE_{elstat} term represents the quasiclassical electrostatic interaction between the unperturbed charge distributions of the prepared fragments, the Pauli repulsion ΔE_{Pauli} corresponds to the energy change associated with the transformation from the superposition of the unperturbed electron densities of the isolated fragments to the wavefunction, which properly obeys the Pauli principle through explicit antisymmetrization and renormalization of the production wavefunction. Since we used -D3(BJ), it gives the dispersion contribution (ΔE_{disp}) also. The orbital term ΔE_{orb} is originated from the mixing of orbitals, charge transfer and polarization between the isolated fragments, which can be further decomposed into contributions from each irreducible representation of the point group of the interacting system as follows:

$$\Delta E_{orb} = \sum_{r} \Delta E_{r} \qquad (2)$$

The combination of the EDA with NOCV enables the partition of the total orbital interactions into pairwise contributions of the orbital interactions which is very vital to get a complete picture of the bonding. The charge deformation $\Delta\rho_k(r)$, resulting from the mixing of the orbital pairs $\psi_k(r)$ and $\psi_{-k}(r)$ of the interacting fragments presents the amount and the shape of the charge flow due to the orbital interactions (Equation 3), and the associated energy term ΔE_{orb} provides with the size of stabilizing orbital energy originated from such interaction (Equation 4).

$$\Delta\rho_{orb}(r) = \sum_{k} \Delta\rho_k(r) = \sum_{k=1}^{N/2} \nu_k [-\psi_{-k}^2(r) + \psi_k^2(r)] \qquad (3)$$

$$\Delta E_{orb} = \sum \Delta E_k^{orb} = \sum^{N/2} \nu_k [-F_{-k,-k}^{TS} + F_{k,k}^{TS}] \qquad (4)$$

More information about the EDA-NOCV method and its application can be obtained in recent reviews articles.[19-21]

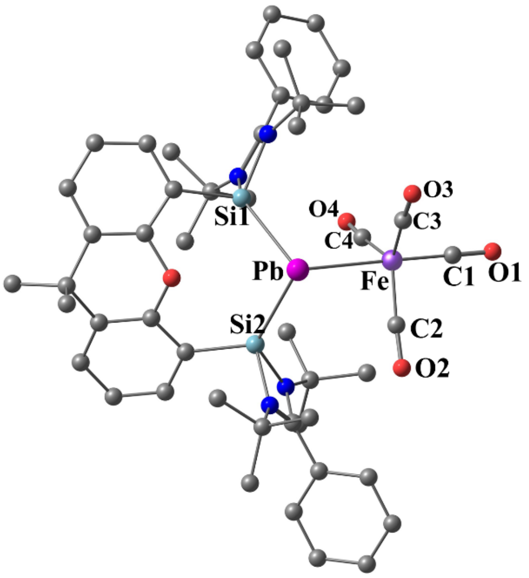

Si1-Pb: 2.759 (2.795)
Si2-Pb: 2.750 (2.791)
Pb-Fe: 2.736 (2.737)
Fe-C1: 1.761 (1.757)
Fe-C2: 1.767 (1.768)
Fe-C3: 1.782 (1.788)
Fe-C4: 1.763 (1.767)
C1-O1: 1.166 (1.161)
C2-O2: 1.178 (1.164)
C3-O3: 1.172 (1.149)
C4-O4: 1.182 (1.161)
<Si1PbSi2: 90.7 (97.6)
<Si1PbFe: 104.5 (106.2)
<Si2PbFe: 103.3 (102.8)

4 (C_1, ^1A)

4 → **3** + $Fe(CO)_4$: D_e (ΔG^{298}) = 84.4 (63.4) kcal/mol

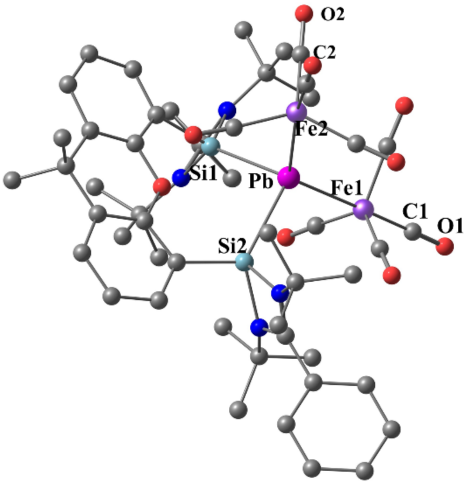

Si1-Pb: 2.748 (2.795)
Si2-Pb: 2.759 (2.791)
Pb-Fe1: 2.646 (2.737)
Pb-Fe2: 2.673
Fe-C1: 1.762-1.790
Fe-C2: 1.754-1.787
C1-O1: 1.163-1.178 (C-O bonds linked to Fe1)
C2-O2: 1.163-1.171 (C-O bonds linked to Fe2)
<Si1PbSi2: 99.5
<Si1PbFe2: 108.3
<Si2PbFe1: 107.5
<Fe2PbFe1: 126.4

3[Fe(CO)$_4$]$_2$ → **4** + $Fe(CO)_4$: D_e (ΔG^{298}) = 54.5 (36.2) kcal/mol

Figure S23. The minimum energy geometry of **4** and **3 [Fe(CO)$_4$]$_2$** at the BP86-D3(BJ)/def2-TZVP level. The computed (experimental) distances and angles are in Å and in degree, respectively.

Table S9. Numerical EDA-NOCV results of complex **3** considering singlet **1** as one fragment and Pb in an excited singlet state as secomd fragment at the BP86-D3(BJ)/TZ2P-ZORA//BP86-D3(BJ)/def2-TZVP level.

**Energy**	**Interaction**	Pb [S, $6s^2 6p_\sigma^0 6p_{\pi\perp}^2$] + [**1**] [S]
ΔE_{int}		-107.6
ΔE_{Pauli}		240.3
ΔE_{disp}[a]		-21.0 (6.0%)
ΔE_{elstat}[a]		-184.8 (53.1%)
ΔE_{orb}[a]		-142.1 (40.8%)
$\Delta E_{orb(1)}$[b]	L→E(p_σ)←L (+,+) σ-donation/sp_σ hybridization	-56.8 (40.0%)
$\Delta E_{orb(2)}$[b]	L→E($p_{\pi\parallel}$)←L (+,-) σ-donation	-43.1 (30.3%)
$\Delta E_{orb(3)}$[b]	L←E($p_{\pi\perp}$)→L π-backdonation	-31.4 (22.1%)
$\Delta E_{orb(rest)}$[b]		-10.8 (7.6%)

[a]The values in parentheses are the percentage contributions to the total attractive interactions (ΔE_{elstat} + ΔE_{orb} + ΔE_{disp}). [b]The values in parentheses are the percentage contributions to the total orbital term, ΔE_{orb}.

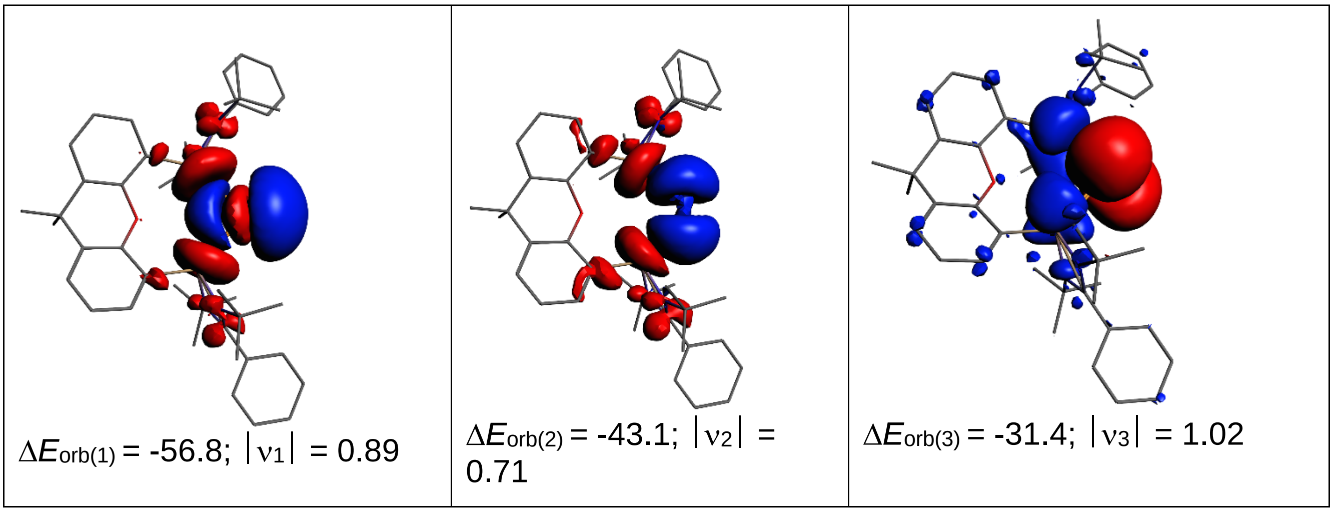

Figure S24. The plot of deformation densities, $\Delta\rho_{(1)-(3)}$ corresponding to the orbital interactions $\Delta E_{orb(1)-(3)}$ in complex **3**. (Table 3). ΔE_{orb} values are given in kcal/mol. Electron density shifted from red to blue region. ν represents the charge eigenvalues. The isosurface values are 0.001 au.

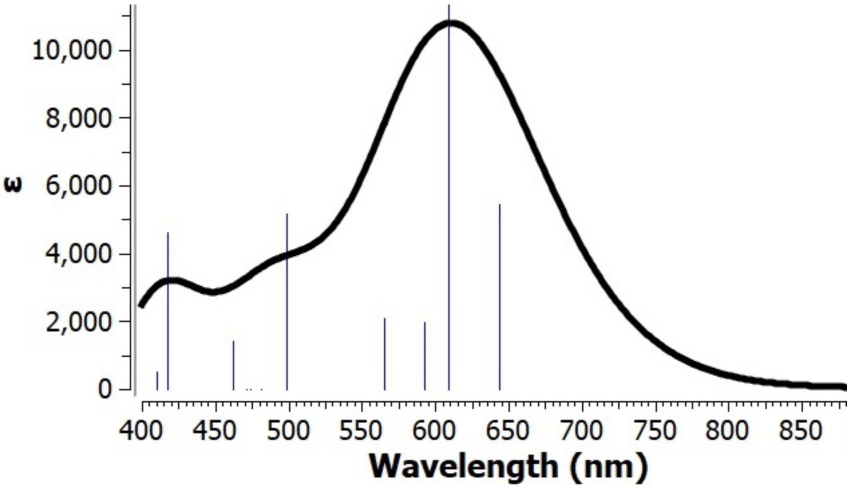

Figure S25. Simulated UV/Vis spectrum of compound **3** at the CAM-B3LYP-D3(BJ)/def2-TZVP//BP86-D3(BJ)/def2-TZVP level.

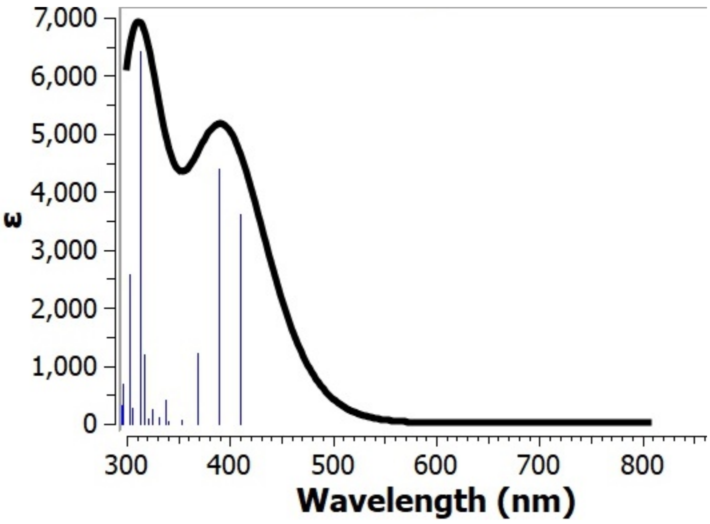

Figure S26. Simulated UV/Vis spectrum of compound **4** at the CAM-B3LYP-D3(BJ)/def2-TZVP//BP86-D3(BJ)/def2-TZVP level.

Table S10. The bond dissociation energies (D_e, kcal/mol) and free energy changes (ΔG^{298K}, kcal/mol) at 298 K computed at the BP86-D3(BJ)/def2-TZVP level.

Process	D_e	ΔG^{298K}
**[3-H]$^+$** $\rightarrow$ **3** + H^+	282.5	277.0
**[3-H$_2$]$^{2+}$** $\rightarrow$ **[3-H]$^+$** + H^+	181.1	176.7
**[3-BH$_3$]** $\rightarrow$ **3** + BH_3	35.5	21.7
**[3-(BH$_3$)$_2$]** $\rightarrow$ **[3-BH$_3$]** + BH_3	28.8	18.0
**[3-(Fe(CO)$_4$)] (4)** $\rightarrow$ **3** + $Fe(CO)_4$	84.4	63.4
**[3-(Fe(CO)$_4$)$_2$]** $\rightarrow$ **4** + $Fe(CO)_4$	54.5	36.2

Table S11. The Cartesian coordinates of the studied complexes at the BP86-D3(BJ)/def2-TZVP level.

3

E = -2817.1142443 au

Pb	0.019472000	-1.334060000	-1.045792000
Si	-1.969588000	0.256089000	-0.378140000
Si	1.939772000	0.275965000	-0.246124000
O	-0.076645000	2.332183000	0.435907000
N	-3.063755000	-0.319521000	1.057616000
N	3.556237000	-0.358357000	-0.955039000
N	-3.564071000	-0.478488000	-1.027253000
N	3.057413000	-0.391553000	1.133613000
C	2.205285000	2.141474000	-0.103997000
C	1.116585000	2.977851000	0.163934000
C	-2.491319000	4.936190000	-0.463907000
H	-2.593813000	6.020928000	-0.510828000
C	-2.269909000	2.120269000	-0.380630000
C	-3.942815000	-0.865699000	0.214562000
C	-1.227829000	2.969351000	0.005153000
C	1.171684000	4.376461000	0.166045000
C	-5.036250000	-1.797339000	0.569580000
C	-3.998008000	-0.965510000	-2.352672000
C	3.951963000	-0.835355000	0.244104000
C	4.055306000	-0.625057000	-2.317529000
C	-3.570023000	4.127826000	-0.842948000
H	-4.497953000	4.593122000	-1.181765000
C	-3.465554000	2.735532000	-0.792134000
H	-4.311336000	2.112054000	-1.090179000
C	-1.292412000	4.366249000	-0.012297000
C	2.858943000	-0.673365000	2.562585000
C	-6.360893000	-1.349811000	0.686044000
H	-6.591576000	-0.301827000	0.489470000
C	3.441161000	2.772039000	-0.338318000
H	4.322779000	2.159101000	-0.539037000
C	-0.096882000	5.149619000	0.535477000
C	5.094686000	-1.738443000	0.512435000
C	-0.211093000	5.172285000	2.085310000
H	-0.237952000	4.150940000	2.489534000
H	-1.133303000	5.689498000	2.390283000
H	0.653055000	5.695260000	2.522079000
C	2.415551000	4.960405000	-0.110893000
H	2.513393000	6.046319000	-0.132829000
C	-7.368193000	-2.237238000	1.063820000
H	-8.395327000	-1.880933000	1.159173000
C	6.364137000	-1.218488000	0.808016000
H	6.507505000	-0.137207000	0.831466000
C	-2.907292000	-0.366792000	2.517982000
C	3.543123000	4.164564000	-0.348005000
H	4.505357000	4.640104000	-0.548850000
C	2.313399000	-2.102054000	2.743938000
H	3.067187000	-2.848241000	2.456722000
H	1.420994000	-2.241971000	2.114909000
H	2.042028000	-2.276393000	3.796349000
C	4.903814000	-3.128639000	0.485346000
H	3.914951000	-3.529099000	0.256497000
C	-3.290960000	-0.075089000	-3.386200000
H	-3.555411000	-0.400336000	-4.402642000
H	-2.197809000	-0.137693000	-3.270004000
H	-3.589224000	0.976300000	-3.265596000

C	-4.732648000	-3.143134000	0.830388000
H	-3.701006000	-3.486228000	0.735144000
C	-7.063847000	-3.578745000	1.322197000
H	-7.853381000	-4.271145000	1.618699000
C	-0.065168000	6.591299000	0.014376000
H	0.784176000	7.140514000	0.443644000
H	-0.975022000	7.130709000	0.311866000
H	0.016701000	6.621435000	-1.081503000
C	-4.257667000	-0.348836000	3.248218000
H	-4.871388000	0.498770000	2.909881000
H	-4.079936000	-0.235525000	4.327969000
H	-4.824584000	-1.275248000	3.094030000
C	-5.745299000	-4.029154000	1.201122000
H	-5.503163000	-5.075147000	1.397160000
C	7.429155000	-2.079389000	1.071758000
H	8.413255000	-1.667709000	1.302128000
C	4.155302000	-0.476850000	3.362261000
H	3.938436000	-0.562971000	4.437475000
H	4.576634000	0.521311000	3.173119000
H	4.910676000	-1.231076000	3.107470000
C	3.354017000	0.389207000	-3.234770000
H	3.616733000	1.418937000	-2.953633000
H	2.260603000	0.280986000	-3.164010000
H	3.657318000	0.220990000	-4.278110000
C	-5.518901000	-0.826518000	-2.527095000
H	-5.841756000	0.200673000	-2.301425000
H	-6.071731000	-1.518862000	-1.880290000
H	-5.787859000	-1.048651000	-3.570305000
C	5.973332000	-3.986564000	0.747983000
H	5.818048000	-5.066533000	0.722773000
C	-3.568272000	-2.430247000	-2.548707000
H	-3.832287000	-2.773971000	-3.560131000
H	-4.067659000	-3.086537000	-1.822528000
H	-2.479350000	-2.527581000	-2.423020000
C	7.237087000	-3.465319000	1.042267000
H	8.071316000	-4.137482000	1.249503000
C	1.802924000	0.338709000	3.033769000
H	0.884707000	0.225509000	2.437532000
H	2.166837000	1.369100000	2.914238000
H	1.555582000	0.167627000	4.090914000
C	5.576138000	-0.423472000	-2.407980000
H	5.889142000	-0.486613000	-3.460556000
H	6.127282000	-1.186393000	-1.844258000
H	5.858944000	0.568726000	-2.026076000
C	-2.110567000	0.894573000	2.891166000
H	-1.160926000	0.929365000	2.337480000
H	-1.883780000	0.894863000	3.966744000
H	-2.680163000	1.802063000	2.646177000
C	-2.096825000	-1.619444000	2.901117000
H	-2.666133000	-2.533076000	2.678963000
H	-1.863870000	-1.610511000	3.976910000
H	-1.159305000	-1.648255000	2.326264000
C	3.675964000	-2.054275000	-2.744984000
H	2.586766000	-2.194312000	-2.672343000
H	4.173149000	-2.799166000	-2.108404000
H	3.980921000	-2.234853000	-3.786771000

References

[1] Y. Wang, A. Kostenko, S. Yao, M. Driess, *J. Am. Chem. Soc.* **2017**, *139*, 13499–13506.
[2] J. A. Gladysz, W. Tam, *J. Org. Chem.* **1978**, *43*, 2279–2280.
[3] G. M. Sheldrick, SHELX-97 Program for Crystal Structure Determination, Universität Göttingen (Germany), **1997**.
[4] A. D. Becke, *Phys. Rev. A* **1988**, *38*, 3098.
[5] J. P. Perdew, *Phys. Rev. B* **1986**, *33*, 8822.
[6] S. Grimme, S. Ehrlich, L. Goerigk, *J. Comput. Chem.* **2011**, *32*, 1456–1465.
[7] S. Grimme, J. Antony, S. Ehrlich, H. Krieg, *J. Chem. Phys.* **2010**, *132*, 154104.
[8] E. D. Glendening, C. R. Landis, F. Weinhold, *J. Comput. Chem.* **2013**, *34*, 1429–1437.
[9] T. Yanai, D. P. Tew, N. C. Handy, *Chem. Phys. Lett.* **2004**, *393*, 51–57.
[10] M. J. Frisch, G. W. Trucks, H. B. Schlegel, G. E. Scuseria, M. A. Robb, J. R. Cheeseman, G. Scalmani, V. Barone, G. A. Petersson, H. Nakatsuji, *Gaussian 16, Revision A.03; Gaussian, Inc., Wallingford CT, 2016.*
[11] T. Ziegler, A. Rauk, *Theor. Chim. acta 1977 461* **1977**, *46*, 1–10.
[12] M. Mitoraj, A. Michalak, *Organometallics* **2007**, *26*, 6576–6580.
[13] M. Mitoraj, A. Michalak, *J. Mol. Model.* **2008**, *14*, 681–687.
[14] ADF2018, SCM, Theoretical chemistry, Vrije Universiteit, Amsterdam, The Netherlands, http://www.scm.com..
[15] G. te Velde, F. M. Bickelhaupt, E. J. Baerends, C. Fonseca Guerra, S. J. A. van Gisbergen, J. G. Snijders, T. Ziegler, *J. Comput. Chem.* **2001**, *22*, 931–967.
[16] A. Michalak, M. Mitoraj, T. Ziegler, *J. Phys. Chem. A* **2008**, *112*, 1933–1939.
[17] M. P. Mitoraj, A. Michalak, T. Ziegler, *J. Chem. Theory Comput.* **2009**, *5*, 962–975.
[18] E. Van Lenthe, E. J. Baerends, *J. Comput. Chem.* **2003**, *24*, 1142–1156.
[19] L. Zhao, M. von Hopffgarten, D. M. Andrada, G. Frenking, *Wiley Interdiscip. Rev. Comput. Mol. Sci.* **2018**, *8*, e1345.
[20] L. Zhao, M. Hermann, W. H. E. Schwarz, G. Frenking, *Nat. Rev. Chem. 2018 31* **2019**, *3*, 48–63.
